# Supplementary material for: Pyruvate Dehydrogenase Kinase 1 inhibition mediated oxidative phosphorylation enhancement in cartilage promotes osteoarthritis progression
Source: BMC Musculoskelet Disord. 2023 Jul 20;24:597. doi: 10.1186/s12891-023-06585-6 (PMC10357736; doi:10.1186/s12891-023-06585-6)
Supplement: Supplementary file 1 — Additional file 1. [file 12891_2023_6585_MOESM1_ESM.zip › MF.Gsea.1653627316242/gsea_report_for_normal_1653627316242.html]

Report for normal 1653627316242 [GSEA]

| GS  follow link to MSigDB | GS DETAILS | SIZE | ES | NES | NOM p-val | FDR q-val | FWER p-val | RANK AT MAX | LEADING EDGE || 1 | GOMF\_MRNA\_BINDING | Details ... | 251 | -0.45 | -2.23 | 0.000 | 0.001 | 0.001 | 2761 | tags=41%, list=21%, signal=51% |
| 2 | GOMF\_MRNA\_3\_UTR\_BINDING | Details ... | 77 | -0.50 | -2.10 | 0.000 | 0.008 | 0.016 | 2757 | tags=42%, list=21%, signal=53% |
| 3 | GOMF\_ATP\_DEPENDENT\_ACTIVITY\_ACTING\_ON\_RNA | Details ... | 63 | -0.51 | -2.04 | 0.000 | 0.012 | 0.034 | 2775 | tags=46%, list=22%, signal=58% |
| 4 | GOMF\_TAU\_PROTEIN\_KINASE\_ACTIVITY | Details ... | 20 | -0.67 | -2.04 | 0.000 | 0.009 | 0.035 | 3312 | tags=70%, list=26%, signal=94% |
| 5 | GOMF\_HELICASE\_ACTIVITY | Details ... | 125 | -0.43 | -1.94 | 0.000 | 0.034 | 0.146 | 3030 | tags=45%, list=23%, signal=58% |
| 6 | GOMF\_STRUCTURAL\_CONSTITUENT\_OF\_RIBOSOME | Details ... | 129 | -0.42 | -1.92 | 0.000 | 0.032 | 0.162 | 2377 | tags=40%, list=18%, signal=48% |
| 7 | GOMF\_TRANSCRIPTION\_COREGULATOR\_BINDING | Details ... | 96 | -0.44 | -1.89 | 0.000 | 0.041 | 0.232 | 3867 | tags=52%, list=30%, signal=74% |
| 8 | GOMF\_TRANSCRIPTION\_COACTIVATOR\_BINDING | Details ... | 36 | -0.52 | -1.86 | 0.000 | 0.048 | 0.301 | 3867 | tags=67%, list=30%, signal=95% |
| 9 | GOMF\_AROMATASE\_ACTIVITY | Details ... | 23 | -0.57 | -1.81 | 0.004 | 0.084 | 0.508 | 4995 | tags=70%, list=39%, signal=113% |
| 10 | GOMF\_UBIQUITIN\_PROTEIN\_TRANSFERASE\_REGULATOR\_ACTIVITY | Details ... | 20 | -0.59 | -1.80 | 0.004 | 0.080 | 0.530 | 2906 | tags=70%, list=23%, signal=90% |
| 11 | GOMF\_MRNA\_5\_UTR\_BINDING | Details ... | 22 | -0.56 | -1.79 | 0.003 | 0.081 | 0.567 | 2761 | tags=59%, list=21%, signal=75% |
| 12 | GOMF\_NUCLEAR\_LOCALIZATION\_SEQUENCE\_BINDING | Details ... | 23 | -0.57 | -1.79 | 0.005 | 0.076 | 0.579 | 2543 | tags=52%, list=20%, signal=65% |
| 13 | GOMF\_PROTEIN\_SERINE\_KINASE\_ACTIVITY | Details ... | 295 | -0.35 | -1.78 | 0.000 | 0.078 | 0.604 | 4153 | tags=48%, list=32%, signal=69% |
| 14 | GOMF\_PROTEIN\_SERINE\_THREONINE\_KINASE\_ACTIVITY | Details ... | 351 | -0.35 | -1.78 | 0.000 | 0.073 | 0.604 | 3776 | tags=42%, list=29%, signal=58% |
| 15 | GOMF\_PHOSPHATIDYLINOSITOL\_BISPHOSPHATE\_PHOSPHATASE\_ACTIVITY | Details ... | 22 | -0.56 | -1.78 | 0.000 | 0.072 | 0.620 | 2151 | tags=36%, list=17%, signal=44% |
| 16 | GOMF\_PROTEIN\_SERINE\_THREONINE\_TYROSINE\_KINASE\_ACTIVITY | Details ... | 375 | -0.34 | -1.78 | 0.000 | 0.067 | 0.622 | 4153 | tags=46%, list=32%, signal=66% |
| 17 | GOMF\_DNA\_BINDING\_TRANSCRIPTION\_REPRESSOR\_ACTIVITY | Details ... | 228 | -0.36 | -1.76 | 0.000 | 0.077 | 0.698 | 2934 | tags=35%, list=23%, signal=44% |
| 18 | GOMF\_NEUTRAL\_AMINO\_ACID\_TRANSMEMBRANE\_TRANSPORTER\_ACTIVITY | Details ... | 32 | -0.51 | -1.75 | 0.003 | 0.081 | 0.740 | 3363 | tags=56%, list=26%, signal=76% |
| 19 | GOMF\_MRNA\_3\_UTR\_AU\_RICH\_REGION\_BINDING | Details ... | 23 | -0.54 | -1.75 | 0.003 | 0.077 | 0.741 | 2629 | tags=43%, list=20%, signal=55% |
| 20 | GOMF\_DNA\_BINDING\_TRANSCRIPTION\_FACTOR\_BINDING | Details ... | 406 | -0.34 | -1.75 | 0.000 | 0.075 | 0.745 | 2302 | tags=27%, list=18%, signal=32% |
| 21 | GOMF\_OXIDOREDUCTASE\_ACTIVITY\_ACTING\_ON\_PAIRED\_DONORS\_WITH\_INCORPORATION\_OR\_REDUCTION\_OF\_MOLECULAR\_OXYGEN\_REDUCED\_FLAVIN\_OR\_FLAVOPROTEIN\_AS\_ONE\_DONOR\_AND\_INCORPORATION\_OF\_ONE\_ATOM\_OF\_OXYGEN | Details ... | 34 | -0.49 | -1.74 | 0.007 | 0.080 | 0.787 | 5053 | tags=65%, list=39%, signal=106% |
| 22 | GOMF\_PROTEIN\_DEMETHYLASE\_ACTIVITY | Details ... | 26 | -0.53 | -1.74 | 0.007 | 0.079 | 0.795 | 1966 | tags=42%, list=15%, signal=50% |
| 23 | GOMF\_NEUROPEPTIDE\_BINDING | Details ... | 22 | -0.54 | -1.70 | 0.012 | 0.106 | 0.899 | 1051 | tags=27%, list=8%, signal=30% |
| 24 | GOMF\_PROTEIN\_KINASE\_ACTIVITY | Details ... | 479 | -0.32 | -1.69 | 0.000 | 0.108 | 0.908 | 3846 | tags=39%, list=30%, signal=54% |
| 25 | GOMF\_14\_3\_3\_PROTEIN\_BINDING | Details ... | 26 | -0.52 | -1.69 | 0.007 | 0.110 | 0.918 | 2380 | tags=38%, list=18%, signal=47% |
| 26 | GOMF\_HISTONE\_BINDING | Details ... | 185 | -0.36 | -1.68 | 0.000 | 0.115 | 0.937 | 2893 | tags=37%, list=22%, signal=47% |
| 27 | GOMF\_POLY\_PYRIMIDINE\_TRACT\_BINDING | Details ... | 20 | -0.55 | -1.67 | 0.005 | 0.116 | 0.942 | 1662 | tags=35%, list=13%, signal=40% |
| 28 | GOMF\_MAP\_KINASE\_KINASE\_KINASE\_ACTIVITY | Details ... | 23 | -0.53 | -1.66 | 0.012 | 0.121 | 0.954 | 4810 | tags=74%, list=37%, signal=118% |
| 29 | GOMF\_INOSITOL\_PHOSPHATE\_PHOSPHATASE\_ACTIVITY | Details ... | 17 | -0.56 | -1.65 | 0.012 | 0.131 | 0.967 | 1325 | tags=29%, list=10%, signal=33% |
| 30 | GOMF\_BICARBONATE\_TRANSMEMBRANE\_TRANSPORTER\_ACTIVITY | Details ... | 15 | -0.58 | -1.65 | 0.022 | 0.131 | 0.975 | 3641 | tags=53%, list=28%, signal=74% |
| 31 | GOMF\_PROTEIN\_PHOSPHATASE\_2A\_BINDING | Details ... | 23 | -0.52 | -1.64 | 0.017 | 0.138 | 0.981 | 2725 | tags=39%, list=21%, signal=50% |
| 32 | GOMF\_GTPASE\_BINDING | Details ... | 240 | -0.33 | -1.63 | 0.000 | 0.144 | 0.985 | 3305 | tags=38%, list=26%, signal=50% |
| 33 | GOMF\_MIRNA\_BINDING | Details ... | 27 | -0.49 | -1.62 | 0.019 | 0.143 | 0.986 | 2757 | tags=44%, list=21%, signal=56% |
| 34 | GOMF\_PHOSPHATIDYLINOSITOL\_PHOSPHATE\_PHOSPHATASE\_ACTIVITY | Details ... | 30 | -0.48 | -1.62 | 0.012 | 0.140 | 0.987 | 2151 | tags=33%, list=17%, signal=40% |
| 35 | GOMF\_SMAD\_BINDING | Details ... | 71 | -0.39 | -1.62 | 0.008 | 0.138 | 0.988 | 2262 | tags=28%, list=18%, signal=34% |
| 36 | GOMF\_METHYLATED\_HISTONE\_BINDING | Details ... | 60 | -0.41 | -1.61 | 0.008 | 0.144 | 0.990 | 2095 | tags=30%, list=16%, signal=36% |
| 37 | GOMF\_CHROMATIN\_BINDING | Details ... | 475 | -0.31 | -1.61 | 0.000 | 0.145 | 0.990 | 3110 | tags=32%, list=24%, signal=41% |
| 38 | GOMF\_ORGANIC\_ANION\_TRANSMEMBRANE\_TRANSPORTER\_ACTIVITY | Details ... | 137 | -0.35 | -1.60 | 0.003 | 0.156 | 0.996 | 3004 | tags=33%, list=23%, signal=42% |
| 39 | GOMF\_RNA\_POLYMERASE\_BINDING | Details ... | 44 | -0.43 | -1.60 | 0.016 | 0.155 | 0.996 | 3055 | tags=48%, list=24%, signal=62% |
| 40 | GOMF\_CORE\_PROMOTER\_SEQUENCE\_SPECIFIC\_DNA\_BINDING | Details ... | 42 | -0.43 | -1.59 | 0.012 | 0.156 | 0.998 | 2271 | tags=36%, list=18%, signal=43% |
| 41 | GOMF\_ARACHIDONIC\_ACID\_MONOOXYGENASE\_ACTIVITY | Details ... | 16 | -0.55 | -1.59 | 0.027 | 0.157 | 0.998 | 5053 | tags=75%, list=39%, signal=123% |
| 42 | GOMF\_PRE\_MRNA\_BINDING | Details ... | 28 | -0.48 | -1.59 | 0.027 | 0.153 | 0.998 | 2557 | tags=46%, list=20%, signal=58% |
| 43 | GOMF\_E\_BOX\_BINDING | Details ... | 42 | -0.43 | -1.59 | 0.012 | 0.153 | 0.998 | 1714 | tags=29%, list=13%, signal=33% |
| 44 | GOMF\_L\_AMINO\_ACID\_TRANSMEMBRANE\_TRANSPORTER\_ACTIVITY | Details ... | 43 | -0.43 | -1.59 | 0.010 | 0.150 | 0.998 | 4838 | tags=65%, list=38%, signal=104% |
| 45 | GOMF\_VITAMIN\_TRANSMEMBRANE\_TRANSPORTER\_ACTIVITY | Details ... | 25 | -0.49 | -1.58 | 0.015 | 0.154 | 0.998 | 4250 | tags=56%, list=33%, signal=83% |
| 46 | GOMF\_METALLOENDOPEPTIDASE\_ACTIVITY | Details ... | 89 | -0.37 | -1.57 | 0.005 | 0.161 | 0.999 | 4451 | tags=48%, list=35%, signal=73% |
| 47 | GOMF\_HSP90\_PROTEIN\_BINDING | Details ... | 40 | -0.42 | -1.57 | 0.011 | 0.158 | 0.999 | 1914 | tags=35%, list=15%, signal=41% |
| 48 | GOMF\_PROTEIN\_TYROSINE\_PHOSPHATASE\_ACTIVITY | Details ... | 86 | -0.37 | -1.57 | 0.014 | 0.156 | 0.999 | 2190 | tags=29%, list=17%, signal=35% |
| 49 | GOMF\_PHOSPHATIDYLINOSITOL\_3\_4\_BISPHOSPHATE\_BINDING | Details ... | 24 | -0.49 | -1.57 | 0.029 | 0.156 | 0.999 | 2008 | tags=33%, list=16%, signal=39% |
| 50 | GOMF\_DEMETHYLASE\_ACTIVITY | Details ... | 33 | -0.45 | -1.57 | 0.014 | 0.154 | 0.999 | 1966 | tags=36%, list=15%, signal=43% |
| 51 | GOMF\_AMINO\_ACID\_TRANSMEMBRANE\_TRANSPORTER\_ACTIVITY | Details ... | 61 | -0.39 | -1.56 | 0.011 | 0.152 | 0.999 | 3363 | tags=39%, list=26%, signal=53% |
| 52 | GOMF\_RIBOSOMAL\_SMALL\_SUBUNIT\_BINDING | Details ... | 15 | -0.56 | -1.56 | 0.031 | 0.152 | 0.999 | 402 | tags=27%, list=3%, signal=27% |
| 53 | GOMF\_AMINO\_ACID\_CATION\_SYMPORTER\_ACTIVITY | Details ... | 18 | -0.51 | -1.56 | 0.041 | 0.157 | 1.000 | 5463 | tags=83%, list=42%, signal=144% |
| 54 | GOMF\_DNA\_HELICASE\_ACTIVITY | Details ... | 58 | -0.39 | -1.55 | 0.018 | 0.156 | 1.000 | 3026 | tags=45%, list=23%, signal=58% |
| 55 | GOMF\_DNA\_BINDING\_TRANSCRIPTION\_ACTIVATOR\_ACTIVITY | Details ... | 371 | -0.30 | -1.55 | 0.000 | 0.158 | 1.000 | 3114 | tags=30%, list=24%, signal=38% |
| 56 | GOMF\_STRUCTURAL\_CONSTITUENT\_OF\_CYTOSKELETON | Details ... | 96 | -0.36 | -1.55 | 0.002 | 0.159 | 1.000 | 2431 | tags=24%, list=19%, signal=29% |
| 57 | GOMF\_RNA\_POLYMERASE\_II\_SPECIFIC\_DNA\_BINDING\_TRANSCRIPTION\_FACTOR\_BINDING | Details ... | 308 | -0.31 | -1.54 | 0.000 | 0.161 | 1.000 | 2302 | tags=25%, list=18%, signal=30% |
| 58 | GOMF\_ATP\_HYDROLYSIS\_ACTIVITY | Details ... | 257 | -0.31 | -1.54 | 0.001 | 0.163 | 1.000 | 3119 | tags=34%, list=24%, signal=44% |
| 59 | GOMF\_LYSINE\_N\_METHYLTRANSFERASE\_ACTIVITY | Details ... | 41 | -0.42 | -1.54 | 0.017 | 0.162 | 1.000 | 2104 | tags=34%, list=16%, signal=41% |
| 60 | GOMF\_SINGLE\_STRANDED\_RNA\_BINDING | Details ... | 70 | -0.38 | -1.53 | 0.008 | 0.164 | 1.000 | 1662 | tags=27%, list=13%, signal=31% |
| 61 | GOMF\_PROTEIN\_SERINE\_THREONINE\_KINASE\_ACTIVATOR\_ACTIVITY | Details ... | 44 | -0.41 | -1.53 | 0.019 | 0.166 | 1.000 | 2970 | tags=41%, list=23%, signal=53% |
| 62 | GOMF\_G\_PROTEIN\_COUPLED\_AMINE\_RECEPTOR\_ACTIVITY | Details ... | 37 | -0.43 | -1.52 | 0.024 | 0.176 | 1.000 | 3111 | tags=46%, list=24%, signal=60% |
| 63 | GOMF\_WIDE\_PORE\_CHANNEL\_ACTIVITY | Details ... | 18 | -0.51 | -1.52 | 0.055 | 0.177 | 1.000 | 925 | tags=22%, list=7%, signal=24% |
| 64 | GOMF\_P53\_BINDING | Details ... | 58 | -0.39 | -1.52 | 0.015 | 0.178 | 1.000 | 2975 | tags=38%, list=23%, signal=49% |
| 65 | GOMF\_KINASE\_INHIBITOR\_ACTIVITY | Details ... | 60 | -0.38 | -1.51 | 0.023 | 0.176 | 1.000 | 2809 | tags=38%, list=22%, signal=49% |
| 66 | GOMF\_AMIDE\_TRANSMEMBRANE\_TRANSPORTER\_ACTIVITY | Details ... | 29 | -0.44 | -1.51 | 0.036 | 0.175 | 1.000 | 3972 | tags=48%, list=31%, signal=70% |
| 67 | GOMF\_CAMP\_BINDING | Details ... | 17 | -0.52 | -1.51 | 0.036 | 0.172 | 1.000 | 4515 | tags=65%, list=35%, signal=99% |
| 68 | GOMF\_PEPTIDE\_HORMONE\_BINDING | Details ... | 50 | -0.40 | -1.51 | 0.020 | 0.171 | 1.000 | 3318 | tags=38%, list=26%, signal=51% |
| 69 | GOMF\_HISTONE\_LYSINE\_N\_METHYLTRANSFERASE\_ACTIVITY | Details ... | 35 | -0.43 | -1.51 | 0.033 | 0.177 | 1.000 | 2104 | tags=34%, list=16%, signal=41% |
| 70 | GOMF\_NOTCH\_BINDING | Details ... | 17 | -0.51 | -1.50 | 0.056 | 0.178 | 1.000 | 2368 | tags=47%, list=18%, signal=58% |
| 71 | GOMF\_NEUROPEPTIDE\_RECEPTOR\_ACTIVITY | Details ... | 31 | -0.44 | -1.50 | 0.037 | 0.183 | 1.000 | 1051 | tags=19%, list=8%, signal=21% |
| 72 | GOMF\_AMINO\_ACID\_SODIUM\_SYMPORTER\_ACTIVITY | Details ... | 16 | -0.51 | -1.50 | 0.040 | 0.182 | 1.000 | 4745 | tags=69%, list=37%, signal=109% |
| 73 | GOMF\_PROTEIN\_TYROSINE\_SERINE\_THREONINE\_PHOSPHATASE\_ACTIVITY | Details ... | 33 | -0.43 | -1.48 | 0.028 | 0.195 | 1.000 | 1416 | tags=27%, list=11%, signal=31% |
| 74 | GOMF\_CYCLOSPORIN\_A\_BINDING | Details ... | 16 | -0.51 | -1.48 | 0.061 | 0.204 | 1.000 | 1146 | tags=38%, list=9%, signal=41% |
| 75 | GOMF\_PHOSPHOLIPID\_TRANSFER\_ACTIVITY | Details ... | 17 | -0.50 | -1.47 | 0.069 | 0.206 | 1.000 | 3048 | tags=47%, list=24%, signal=62% |
| 76 | GOMF\_PEPTIDE\_N\_ACETYLTRANSFERASE\_ACTIVITY | Details ... | 38 | -0.41 | -1.46 | 0.045 | 0.217 | 1.000 | 1362 | tags=26%, list=11%, signal=29% |
| 77 | GOMF\_ACTIVIN\_BINDING | Details ... | 15 | -0.51 | -1.46 | 0.087 | 0.216 | 1.000 | 2331 | tags=33%, list=18%, signal=41% |
| 78 | GOMF\_CYCLIC\_NUCLEOTIDE\_BINDING | Details ... | 31 | -0.43 | -1.46 | 0.038 | 0.216 | 1.000 | 4515 | tags=58%, list=35%, signal=89% |
| 79 | GOMF\_SEROTONIN\_RECEPTOR\_ACTIVITY | Details ... | 24 | -0.45 | -1.45 | 0.050 | 0.232 | 1.000 | 2896 | tags=46%, list=22%, signal=59% |
| 80 | GOMF\_NUCLEAR\_RECEPTOR\_BINDING | Details ... | 129 | -0.32 | -1.45 | 0.006 | 0.232 | 1.000 | 1756 | tags=21%, list=14%, signal=24% |
| 81 | GOMF\_HISTONE\_DEACETYLASE\_BINDING | Details ... | 105 | -0.33 | -1.44 | 0.017 | 0.241 | 1.000 | 3262 | tags=33%, list=25%, signal=44% |
| 82 | GOMF\_PROMOTER\_SPECIFIC\_CHROMATIN\_BINDING | Details ... | 59 | -0.37 | -1.44 | 0.034 | 0.242 | 1.000 | 2528 | tags=31%, list=20%, signal=38% |
| 83 | GOMF\_PHOSPHATASE\_REGULATOR\_ACTIVITY | Details ... | 80 | -0.35 | -1.44 | 0.020 | 0.240 | 1.000 | 3394 | tags=40%, list=26%, signal=54% |
| 84 | GOMF\_TRANSCRIPTION\_COREGULATOR\_ACTIVITY | Details ... | 401 | -0.28 | -1.44 | 0.000 | 0.239 | 1.000 | 1756 | tags=20%, list=14%, signal=22% |
| 85 | GOMF\_CADHERIN\_BINDING | Details ... | 307 | -0.28 | -1.43 | 0.004 | 0.243 | 1.000 | 3936 | tags=40%, list=31%, signal=56% |
| 86 | GOMF\_ORGANIC\_ACID\_SODIUM\_SYMPORTER\_ACTIVITY | Details ... | 24 | -0.44 | -1.42 | 0.063 | 0.255 | 1.000 | 4745 | tags=71%, list=37%, signal=112% |
| 87 | GOMF\_TRANSCRIPTION\_COREPRESSOR\_ACTIVITY | Details ... | 148 | -0.31 | -1.42 | 0.013 | 0.258 | 1.000 | 4215 | tags=44%, list=33%, signal=64% |
| 88 | GOMF\_GTPASE\_ACTIVATOR\_ACTIVITY | Details ... | 198 | -0.29 | -1.42 | 0.009 | 0.257 | 1.000 | 3072 | tags=31%, list=24%, signal=40% |
| 89 | GOMF\_MODIFICATION\_DEPENDENT\_PROTEIN\_BINDING | Details ... | 125 | -0.31 | -1.42 | 0.014 | 0.256 | 1.000 | 2208 | tags=24%, list=17%, signal=29% |
| 90 | GOMF\_INWARD\_RECTIFIER\_POTASSIUM\_CHANNEL\_ACTIVITY | Details ... | 23 | -0.44 | -1.41 | 0.072 | 0.265 | 1.000 | 4045 | tags=52%, list=31%, signal=76% |
| 91 | GOMF\_ACETYLTRANSFERASE\_ACTIVITY | Details ... | 70 | -0.34 | -1.41 | 0.029 | 0.263 | 1.000 | 1388 | tags=21%, list=11%, signal=24% |
| 92 | GOMF\_REGULATORY\_RNA\_BINDING | Details ... | 35 | -0.40 | -1.40 | 0.063 | 0.267 | 1.000 | 2757 | tags=43%, list=21%, signal=54% |
| 93 | GOMF\_SCAFFOLD\_PROTEIN\_BINDING | Details ... | 63 | -0.36 | -1.40 | 0.048 | 0.266 | 1.000 | 3147 | tags=38%, list=24%, signal=50% |
| 94 | GOMF\_NEUROPEPTIDE\_RECEPTOR\_BINDING | Details ... | 26 | -0.43 | -1.40 | 0.082 | 0.265 | 1.000 | 4148 | tags=50%, list=32%, signal=74% |
| 95 | GOMF\_OXIDOREDUCTASE\_ACTIVITY\_ACTING\_ON\_THE\_CH\_NH\_GROUP\_OF\_DONORS | Details ... | 20 | -0.46 | -1.40 | 0.100 | 0.264 | 1.000 | 822 | tags=25%, list=6%, signal=27% |
| 96 | GOMF\_POLY\_PURINE\_TRACT\_BINDING | Details ... | 23 | -0.44 | -1.40 | 0.088 | 0.262 | 1.000 | 1202 | tags=26%, list=9%, signal=29% |
| 97 | GOMF\_N\_ACETYLTRANSFERASE\_ACTIVITY | Details ... | 49 | -0.36 | -1.39 | 0.044 | 0.273 | 1.000 | 1362 | tags=22%, list=11%, signal=25% |
| 98 | GOMF\_OLFACTORY\_RECEPTOR\_ACTIVITY | Details ... | 40 | -0.38 | -1.38 | 0.067 | 0.286 | 1.000 | 4122 | tags=43%, list=32%, signal=62% |
| 99 | GOMF\_CALCIUM\_DEPENDENT\_PROTEIN\_KINASE\_ACTIVITY | Details ... | 22 | -0.43 | -1.38 | 0.103 | 0.285 | 1.000 | 1437 | tags=23%, list=11%, signal=26% |
| 100 | GOMF\_NUCLEOSIDE\_TRIPHOSPHATASE\_REGULATOR\_ACTIVITY | Details ... | 354 | -0.27 | -1.38 | 0.005 | 0.283 | 1.000 | 2986 | tags=29%, list=23%, signal=37% |
| 101 | GOMF\_ORGANIC\_ACID\_TRANSMEMBRANE\_TRANSPORTER\_ACTIVITY |  | 114 | -0.31 | -1.38 | 0.035 | 0.289 | 1.000 | 3004 | tags=31%, list=23%, signal=40% |
| 102 | GOMF\_HORMONE\_ACTIVITY |  | 95 | -0.32 | -1.37 | 0.032 | 0.297 | 1.000 | 2391 | tags=23%, list=19%, signal=28% |
| 103 | GOMF\_DISORDERED\_DOMAIN\_SPECIFIC\_BINDING |  | 28 | -0.41 | -1.37 | 0.069 | 0.297 | 1.000 | 2842 | tags=39%, list=22%, signal=50% |
| 104 | GOMF\_LYSINE\_ACETYLATED\_HISTONE\_BINDING |  | 22 | -0.43 | -1.37 | 0.096 | 0.299 | 1.000 | 688 | tags=23%, list=5%, signal=24% |
| 105 | GOMF\_SIGNAL\_SEQUENCE\_BINDING |  | 43 | -0.37 | -1.37 | 0.072 | 0.297 | 1.000 | 2783 | tags=42%, list=22%, signal=53% |
| 106 | GOMF\_KINASE\_REGULATOR\_ACTIVITY |  | 191 | -0.29 | -1.37 | 0.018 | 0.298 | 1.000 | 2842 | tags=30%, list=22%, signal=38% |
| 107 | GOMF\_NUCLEAR\_IMPORT\_SIGNAL\_RECEPTOR\_ACTIVITY |  | 17 | -0.47 | -1.36 | 0.125 | 0.301 | 1.000 | 1783 | tags=35%, list=14%, signal=41% |
| 108 | GOMF\_CHROMATIN\_DNA\_BINDING |  | 90 | -0.32 | -1.36 | 0.028 | 0.299 | 1.000 | 3082 | tags=36%, list=24%, signal=46% |
| 109 | GOMF\_SUMO\_TRANSFERASE\_ACTIVITY |  | 17 | -0.46 | -1.36 | 0.119 | 0.298 | 1.000 | 2301 | tags=35%, list=18%, signal=43% |
| 110 | GOMF\_PROTEIN\_TRANSPORTER\_ACTIVITY |  | 24 | -0.42 | -1.36 | 0.089 | 0.296 | 1.000 | 3028 | tags=38%, list=23%, signal=49% |
| 111 | GOMF\_GROWTH\_FACTOR\_BINDING |  | 122 | -0.30 | -1.36 | 0.033 | 0.301 | 1.000 | 2917 | tags=30%, list=23%, signal=39% |
| 112 | GOMF\_MODIFIED\_AMINO\_ACID\_TRANSMEMBRANE\_TRANSPORTER\_ACTIVITY |  | 20 | -0.45 | -1.36 | 0.107 | 0.299 | 1.000 | 3836 | tags=55%, list=30%, signal=78% |
| 113 | GOMF\_PROTEIN\_TYROSINE\_KINASE\_ACTIVITY |  | 131 | -0.30 | -1.35 | 0.041 | 0.300 | 1.000 | 2810 | tags=28%, list=22%, signal=36% |
| 114 | GOMF\_EXOGENOUS\_PROTEIN\_BINDING |  | 74 | -0.33 | -1.35 | 0.063 | 0.300 | 1.000 | 4075 | tags=42%, list=32%, signal=61% |
| 115 | GOMF\_PROTEIN\_PHOSPHORYLATED\_AMINO\_ACID\_BINDING |  | 49 | -0.36 | -1.35 | 0.082 | 0.302 | 1.000 | 2066 | tags=31%, list=16%, signal=36% |
| 116 | GOMF\_CYCLIC\_NUCLEOTIDE\_PHOSPHODIESTERASE\_ACTIVITY |  | 24 | -0.42 | -1.35 | 0.115 | 0.301 | 1.000 | 4105 | tags=58%, list=32%, signal=85% |
| 117 | GOMF\_CHEMOATTRACTANT\_ACTIVITY |  | 28 | -0.40 | -1.35 | 0.095 | 0.300 | 1.000 | 1854 | tags=32%, list=14%, signal=37% |
| 118 | GOMF\_PROTEASE\_BINDING |  | 118 | -0.30 | -1.35 | 0.053 | 0.299 | 1.000 | 3073 | tags=32%, list=24%, signal=42% |
| 119 | GOMF\_SECONDARY\_ACTIVE\_TRANSMEMBRANE\_TRANSPORTER\_ACTIVITY |  | 174 | -0.29 | -1.35 | 0.023 | 0.300 | 1.000 | 3015 | tags=31%, list=23%, signal=40% |
| 120 | GOMF\_MAGNESIUM\_ION\_BINDING |  | 174 | -0.28 | -1.34 | 0.031 | 0.303 | 1.000 | 3512 | tags=37%, list=27%, signal=50% |
| 121 | GOMF\_CYCLIN\_DEPENDENT\_PROTEIN\_SERINE\_THREONINE\_KINASE\_REGULATOR\_ACTIVITY |  | 41 | -0.36 | -1.34 | 0.085 | 0.301 | 1.000 | 2578 | tags=32%, list=20%, signal=39% |
| 122 | GOMF\_HEAT\_SHOCK\_PROTEIN\_BINDING |  | 111 | -0.30 | -1.34 | 0.046 | 0.302 | 1.000 | 3786 | tags=44%, list=29%, signal=62% |
| 123 | GOMF\_PROTEIN\_KINASE\_C\_ACTIVITY |  | 16 | -0.46 | -1.34 | 0.116 | 0.304 | 1.000 | 1437 | tags=25%, list=11%, signal=28% |
| 124 | GOMF\_NF\_KAPPAB\_BINDING |  | 28 | -0.40 | -1.34 | 0.088 | 0.301 | 1.000 | 2182 | tags=36%, list=17%, signal=43% |
| 125 | GOMF\_PHOSPHOLIPID\_TRANSPORTER\_ACTIVITY |  | 47 | -0.35 | -1.34 | 0.086 | 0.303 | 1.000 | 4046 | tags=47%, list=31%, signal=68% |
| 126 | GOMF\_KINASE\_ACTIVATOR\_ACTIVITY |  | 88 | -0.31 | -1.34 | 0.057 | 0.301 | 1.000 | 2323 | tags=26%, list=18%, signal=32% |
| 127 | GOMF\_TAU\_PROTEIN\_BINDING |  | 41 | -0.37 | -1.33 | 0.098 | 0.300 | 1.000 | 2842 | tags=41%, list=22%, signal=53% |
| 128 | GOMF\_NUCLEAR\_RECEPTOR\_ACTIVITY |  | 50 | -0.35 | -1.33 | 0.064 | 0.299 | 1.000 | 4685 | tags=54%, list=36%, signal=84% |
| 129 | GOMF\_COLLAGEN\_BINDING |  | 61 | -0.34 | -1.33 | 0.076 | 0.298 | 1.000 | 2589 | tags=31%, list=20%, signal=39% |
| 130 | GOMF\_POLY\_A\_BINDING |  | 19 | -0.43 | -1.33 | 0.142 | 0.307 | 1.000 | 4529 | tags=63%, list=35%, signal=97% |
| 131 | GOMF\_HORMONE\_BINDING |  | 82 | -0.31 | -1.32 | 0.051 | 0.312 | 1.000 | 3318 | tags=32%, list=26%, signal=42% |
| 132 | GOMF\_SOLUTE\_SODIUM\_SYMPORTER\_ACTIVITY |  | 59 | -0.34 | -1.32 | 0.079 | 0.314 | 1.000 | 4470 | tags=51%, list=35%, signal=77% |
| 133 | GOMF\_PROTEIN\_SERINE\_THREONINE\_PHOSPHATASE\_ACTIVITY |  | 73 | -0.32 | -1.32 | 0.074 | 0.323 | 1.000 | 2151 | tags=26%, list=17%, signal=31% |
| 134 | GOMF\_PROTEIN\_KINASE\_A\_BINDING |  | 42 | -0.36 | -1.31 | 0.090 | 0.329 | 1.000 | 3951 | tags=52%, list=31%, signal=75% |
| 135 | GOMF\_SODIUM\_ION\_TRANSMEMBRANE\_TRANSPORTER\_ACTIVITY |  | 116 | -0.30 | -1.31 | 0.078 | 0.334 | 1.000 | 4745 | tags=48%, list=37%, signal=76% |
| 136 | GOMF\_BETA\_CATENIN\_BINDING |  | 76 | -0.32 | -1.31 | 0.088 | 0.332 | 1.000 | 2573 | tags=29%, list=20%, signal=36% |
| 137 | GOMF\_PEPTIDE\_LYSINE\_N\_ACETYLTRANSFERASE\_ACTIVITY |  | 33 | -0.37 | -1.31 | 0.134 | 0.332 | 1.000 | 1362 | tags=24%, list=11%, signal=27% |
| 138 | GOMF\_RETINOID\_X\_RECEPTOR\_BINDING |  | 15 | -0.46 | -1.30 | 0.149 | 0.334 | 1.000 | 534 | tags=20%, list=4%, signal=21% |
| 139 | GOMF\_GENERAL\_TRANSCRIPTION\_INITIATION\_FACTOR\_ACTIVITY |  | 39 | -0.37 | -1.30 | 0.138 | 0.336 | 1.000 | 2320 | tags=31%, list=18%, signal=37% |
| 140 | GOMF\_STRUCTURAL\_CONSTITUENT\_OF\_EYE\_LENS |  | 22 | -0.42 | -1.30 | 0.149 | 0.337 | 1.000 | 2652 | tags=32%, list=21%, signal=40% |
| 141 | GOMF\_EXTRACELLULAR\_MATRIX\_BINDING |  | 47 | -0.35 | -1.29 | 0.116 | 0.350 | 1.000 | 4141 | tags=51%, list=32%, signal=75% |
| 142 | GOMF\_TRANSLATION\_REGULATOR\_ACTIVITY |  | 110 | -0.29 | -1.29 | 0.063 | 0.350 | 1.000 | 3193 | tags=36%, list=25%, signal=48% |
| 143 | GOMF\_CATION\_CATION\_ANTIPORTER\_ACTIVITY |  | 17 | -0.44 | -1.28 | 0.171 | 0.363 | 1.000 | 710 | tags=24%, list=6%, signal=25% |
| 144 | GOMF\_GLUTAMATE\_RECEPTOR\_ACTIVITY |  | 24 | -0.40 | -1.28 | 0.132 | 0.362 | 1.000 | 1596 | tags=29%, list=12%, signal=33% |
| 145 | GOMF\_TRANSMEMBRANE\_RECEPTOR\_PROTEIN\_KINASE\_ACTIVITY |  | 76 | -0.31 | -1.28 | 0.082 | 0.362 | 1.000 | 2805 | tags=29%, list=22%, signal=37% |
| 146 | GOMF\_PEPTIDE\_RECEPTOR\_ACTIVITY |  | 128 | -0.28 | -1.28 | 0.064 | 0.364 | 1.000 | 4799 | tags=43%, list=37%, signal=68% |
| 147 | GOMF\_SNORNA\_BINDING |  | 26 | -0.39 | -1.28 | 0.135 | 0.367 | 1.000 | 3625 | tags=58%, list=28%, signal=80% |
| 148 | GOMF\_ATP\_DEPENDENT\_ACTIVITY\_ACTING\_ON\_DNA |  | 85 | -0.30 | -1.28 | 0.085 | 0.365 | 1.000 | 2893 | tags=35%, list=22%, signal=45% |
| 149 | GOMF\_CYCLIN\_DEPENDENT\_PROTEIN\_KINASE\_ACTIVITY |  | 26 | -0.39 | -1.27 | 0.148 | 0.366 | 1.000 | 3677 | tags=54%, list=29%, signal=75% |
| 150 | GOMF\_TRANSCRIPTION\_COACTIVATOR\_ACTIVITY |  | 225 | -0.26 | -1.27 | 0.043 | 0.365 | 1.000 | 3315 | tags=31%, list=26%, signal=41% |
| 151 | GOMF\_LRR\_DOMAIN\_BINDING |  | 15 | -0.44 | -1.27 | 0.171 | 0.363 | 1.000 | 1451 | tags=40%, list=11%, signal=45% |
| 152 | GOMF\_STEROID\_HYDROXYLASE\_ACTIVITY |  | 32 | -0.37 | -1.27 | 0.150 | 0.365 | 1.000 | 5053 | tags=63%, list=39%, signal=102% |
| 153 | GOMF\_ENDODEOXYRIBONUCLEASE\_ACTIVITY |  | 32 | -0.36 | -1.27 | 0.153 | 0.366 | 1.000 | 1955 | tags=28%, list=15%, signal=33% |
| 154 | GOMF\_G\_PROTEIN\_COUPLED\_RECEPTOR\_ACTIVITY |  | 361 | -0.25 | -1.27 | 0.035 | 0.367 | 1.000 | 4130 | tags=36%, list=32%, signal=51% |
| 155 | GOMF\_PEPTIDE\_BINDING |  | 280 | -0.25 | -1.26 | 0.044 | 0.373 | 1.000 | 3860 | tags=36%, list=30%, signal=50% |
| 156 | GOMF\_PHOSPHATIDYLINOSITOL\_3\_4\_5\_TRISPHOSPHATE\_BINDING |  | 32 | -0.36 | -1.26 | 0.148 | 0.372 | 1.000 | 2902 | tags=31%, list=22%, signal=40% |
| 157 | GOMF\_GROWTH\_FACTOR\_RECEPTOR\_BINDING |  | 126 | -0.28 | -1.26 | 0.087 | 0.376 | 1.000 | 3209 | tags=31%, list=25%, signal=41% |
| 158 | GOMF\_RIBONUCLEOPROTEIN\_COMPLEX\_BINDING |  | 122 | -0.28 | -1.26 | 0.097 | 0.378 | 1.000 | 1214 | tags=17%, list=9%, signal=19% |
| 159 | GOMF\_PHOSPHATIDYLINOSITOL\_MONOPHOSPHATE\_PHOSPHATASE\_ACTIVITY |  | 19 | -0.41 | -1.26 | 0.169 | 0.376 | 1.000 | 3443 | tags=42%, list=27%, signal=57% |
| 160 | GOMF\_PROTEOGLYCAN\_BINDING |  | 35 | -0.36 | -1.26 | 0.147 | 0.374 | 1.000 | 3531 | tags=43%, list=27%, signal=59% |
| 161 | GOMF\_ENZYME\_ACTIVATOR\_ACTIVITY |  | 424 | -0.24 | -1.25 | 0.028 | 0.379 | 1.000 | 2986 | tags=29%, list=23%, signal=36% |
| 162 | GOMF\_PHOSPHOPROTEIN\_PHOSPHATASE\_ACTIVITY |  | 138 | -0.27 | -1.25 | 0.090 | 0.380 | 1.000 | 2190 | tags=23%, list=17%, signal=28% |
| 163 | GOMF\_CXCR\_CHEMOKINE\_RECEPTOR\_BINDING |  | 18 | -0.41 | -1.25 | 0.190 | 0.379 | 1.000 | 3480 | tags=44%, list=27%, signal=61% |
| 164 | GOMF\_SNRNA\_BINDING |  | 40 | -0.34 | -1.25 | 0.138 | 0.377 | 1.000 | 2557 | tags=33%, list=20%, signal=40% |
| 165 | GOMF\_R\_SMAD\_BINDING |  | 23 | -0.39 | -1.25 | 0.176 | 0.379 | 1.000 | 2567 | tags=39%, list=20%, signal=49% |
| 166 | GOMF\_ORGANIC\_CATION\_TRANSMEMBRANE\_TRANSPORTER\_ACTIVITY |  | 17 | -0.41 | -1.24 | 0.201 | 0.398 | 1.000 | 2962 | tags=41%, list=23%, signal=53% |
| 167 | GOMF\_OXYGEN\_BINDING |  | 32 | -0.36 | -1.24 | 0.178 | 0.396 | 1.000 | 5218 | tags=63%, list=40%, signal=105% |
| 168 | GOMF\_PROTEIN\_PHOSPHATASE\_REGULATOR\_ACTIVITY |  | 65 | -0.31 | -1.24 | 0.139 | 0.401 | 1.000 | 3394 | tags=37%, list=26%, signal=50% |
| 169 | GOMF\_G\_PROTEIN\_COUPLED\_RECEPTOR\_BINDING |  | 228 | -0.26 | -1.24 | 0.059 | 0.400 | 1.000 | 4148 | tags=38%, list=32%, signal=55% |
| 170 | GOMF\_STRUCTURAL\_CONSTITUENT\_OF\_NUCLEAR\_PORE |  | 24 | -0.39 | -1.23 | 0.179 | 0.401 | 1.000 | 2447 | tags=29%, list=19%, signal=36% |
| 171 | GOMF\_SUMO\_BINDING |  | 15 | -0.43 | -1.23 | 0.221 | 0.403 | 1.000 | 3664 | tags=47%, list=28%, signal=65% |
| 172 | GOMF\_SOLUTE\_ANION\_ANTIPORTER\_ACTIVITY |  | 23 | -0.38 | -1.23 | 0.168 | 0.404 | 1.000 | 3641 | tags=39%, list=28%, signal=54% |
| 173 | GOMF\_KINESIN\_BINDING |  | 36 | -0.35 | -1.23 | 0.162 | 0.404 | 1.000 | 2529 | tags=33%, list=20%, signal=41% |
| 174 | GOMF\_GUANYL\_NUCLEOTIDE\_EXCHANGE\_FACTOR\_ACTIVITY |  | 164 | -0.26 | -1.23 | 0.089 | 0.402 | 1.000 | 2923 | tags=28%, list=23%, signal=36% |
| 175 | GOMF\_ANION\_CATION\_SYMPORTER\_ACTIVITY |  | 20 | -0.39 | -1.23 | 0.179 | 0.401 | 1.000 | 4009 | tags=50%, list=31%, signal=72% |
| 176 | GOMF\_METHYL\_CPG\_BINDING |  | 18 | -0.41 | -1.23 | 0.206 | 0.399 | 1.000 | 2940 | tags=33%, list=23%, signal=43% |
| 177 | GOMF\_OXIDOREDUCTASE\_ACTIVITY\_ACTING\_ON\_PAIRED\_DONORS\_WITH\_INCORPORATION\_OR\_REDUCTION\_OF\_MOLECULAR\_OXYGEN |  | 140 | -0.27 | -1.23 | 0.109 | 0.397 | 1.000 | 3320 | tags=33%, list=26%, signal=44% |
| 178 | GOMF\_PROTEIN\_HORMONE\_RECEPTOR\_ACTIVITY |  | 17 | -0.42 | -1.22 | 0.187 | 0.406 | 1.000 | 2443 | tags=29%, list=19%, signal=36% |
| 179 | GOMF\_GENERAL\_TRANSCRIPTION\_INITIATION\_FACTOR\_BINDING |  | 48 | -0.32 | -1.22 | 0.155 | 0.409 | 1.000 | 1960 | tags=27%, list=15%, signal=32% |
| 180 | GOMF\_TRANSMEMBRANE\_RECEPTOR\_PROTEIN\_TYROSINE\_KINASE\_ACTIVITY |  | 60 | -0.31 | -1.22 | 0.163 | 0.410 | 1.000 | 2805 | tags=30%, list=22%, signal=38% |
| 181 | GOMF\_LIPID\_TRANSFER\_ACTIVITY |  | 38 | -0.34 | -1.22 | 0.195 | 0.417 | 1.000 | 3048 | tags=34%, list=24%, signal=45% |
| 182 | GOMF\_EXTRACELLULAR\_MATRIX\_STRUCTURAL\_CONSTITUENT\_CONFERRING\_TENSILE\_STRENGTH |  | 33 | -0.35 | -1.21 | 0.183 | 0.424 | 1.000 | 4118 | tags=48%, list=32%, signal=71% |
| 183 | GOMF\_NUCLEOCYTOPLASMIC\_CARRIER\_ACTIVITY |  | 27 | -0.36 | -1.21 | 0.192 | 0.424 | 1.000 | 3902 | tags=48%, list=30%, signal=69% |
| 184 | GOMF\_TRNA\_BINDING |  | 46 | -0.32 | -1.21 | 0.188 | 0.422 | 1.000 | 2748 | tags=30%, list=21%, signal=39% |
| 185 | GOMF\_MONOOXYGENASE\_ACTIVITY |  | 81 | -0.29 | -1.21 | 0.144 | 0.422 | 1.000 | 3733 | tags=36%, list=29%, signal=50% |
| 186 | GOMF\_CHEMOKINE\_ACTIVITY |  | 40 | -0.34 | -1.21 | 0.180 | 0.422 | 1.000 | 3548 | tags=40%, list=28%, signal=55% |
| 187 | GOMF\_NAD\_P\_PLUS\_NUCLEOSIDASE\_ACTIVITY |  | 15 | -0.43 | -1.21 | 0.239 | 0.425 | 1.000 | 3507 | tags=53%, list=27%, signal=73% |
| 188 | GOMF\_TRANSMEMBRANE\_RECEPTOR\_PROTEIN\_PHOSPHATASE\_ACTIVITY |  | 17 | -0.41 | -1.20 | 0.219 | 0.427 | 1.000 | 1613 | tags=29%, list=13%, signal=34% |
| 189 | GOMF\_PHOSPHATIDYLINOSITOL\_3\_KINASE\_BINDING |  | 21 | -0.39 | -1.20 | 0.219 | 0.430 | 1.000 | 1803 | tags=29%, list=14%, signal=33% |
| 190 | GOMF\_N\_METHYLTRANSFERASE\_ACTIVITY |  | 72 | -0.29 | -1.20 | 0.167 | 0.429 | 1.000 | 2911 | tags=32%, list=23%, signal=41% |
| 191 | GOMF\_PHOSPHATASE\_ACTIVITY |  | 214 | -0.25 | -1.20 | 0.106 | 0.435 | 1.000 | 2024 | tags=21%, list=16%, signal=25% |
| 192 | GOMF\_PHOSPHOPROTEIN\_BINDING |  | 78 | -0.28 | -1.19 | 0.165 | 0.441 | 1.000 | 2247 | tags=26%, list=17%, signal=31% |
| 193 | GOMF\_ANTIPORTER\_ACTIVITY |  | 64 | -0.30 | -1.19 | 0.184 | 0.442 | 1.000 | 1616 | tags=20%, list=13%, signal=23% |
| 194 | GOMF\_METAL\_ION\_TRANSMEMBRANE\_TRANSPORTER\_ACTIVITY |  | 318 | -0.23 | -1.19 | 0.075 | 0.444 | 1.000 | 3035 | tags=28%, list=24%, signal=36% |
| 195 | GOMF\_DEATH\_RECEPTOR\_BINDING |  | 19 | -0.39 | -1.19 | 0.227 | 0.442 | 1.000 | 1005 | tags=26%, list=8%, signal=28% |
| 196 | GOMF\_CATALYTIC\_ACTIVITY\_ACTING\_ON\_A\_NUCLEIC\_ACID |  | 452 | -0.23 | -1.19 | 0.076 | 0.442 | 1.000 | 2893 | tags=28%, list=22%, signal=35% |
| 197 | GOMF\_PHOSPHORIC\_ESTER\_HYDROLASE\_ACTIVITY |  | 287 | -0.24 | -1.19 | 0.115 | 0.443 | 1.000 | 2190 | tags=22%, list=17%, signal=26% |
| 198 | GOMF\_ANION\_TRANSMEMBRANE\_TRANSPORTER\_ACTIVITY |  | 239 | -0.24 | -1.19 | 0.121 | 0.441 | 1.000 | 4410 | tags=41%, list=34%, signal=61% |
| 199 | GOMF\_SOLUTE\_CATION\_SYMPORTER\_ACTIVITY |  | 79 | -0.29 | -1.19 | 0.156 | 0.444 | 1.000 | 4470 | tags=47%, list=35%, signal=71% |
| 200 | GOMF\_DICARBOXYLIC\_ACID\_TRANSMEMBRANE\_TRANSPORTER\_ACTIVITY |  | 23 | -0.37 | -1.18 | 0.231 | 0.448 | 1.000 | 4745 | tags=57%, list=37%, signal=89% |
| 201 | GOMF\_K63\_LINKED\_POLYUBIQUITIN\_MODIFICATION\_DEPENDENT\_PROTEIN\_BINDING |  | 15 | -0.42 | -1.18 | 0.262 | 0.449 | 1.000 | 1916 | tags=33%, list=15%, signal=39% |
| 202 | GOMF\_NUCLEAR\_RECEPTOR\_COACTIVATOR\_ACTIVITY |  | 46 | -0.31 | -1.18 | 0.226 | 0.454 | 1.000 | 3290 | tags=37%, list=26%, signal=49% |
| 203 | GOMF\_HISTONE\_METHYLTRANSFERASE\_ACTIVITY |  | 46 | -0.31 | -1.18 | 0.214 | 0.452 | 1.000 | 2104 | tags=26%, list=16%, signal=31% |
| 204 | GOMF\_CATALYTIC\_ACTIVITY\_ACTING\_ON\_RNA |  | 287 | -0.24 | -1.18 | 0.115 | 0.456 | 1.000 | 2884 | tags=28%, list=22%, signal=36% |
| 205 | GOMF\_EPIDERMAL\_GROWTH\_FACTOR\_RECEPTOR\_BINDING |  | 29 | -0.35 | -1.17 | 0.253 | 0.456 | 1.000 | 2409 | tags=28%, list=19%, signal=34% |
| 206 | GOMF\_INTEGRIN\_BINDING |  | 145 | -0.25 | -1.17 | 0.161 | 0.464 | 1.000 | 4165 | tags=41%, list=32%, signal=60% |
| 207 | GOMF\_PHOSPHATIDIC\_ACID\_BINDING |  | 16 | -0.41 | -1.17 | 0.261 | 0.462 | 1.000 | 4497 | tags=63%, list=35%, signal=96% |
| 208 | GOMF\_NEUROPEPTIDE\_HORMONE\_ACTIVITY |  | 27 | -0.36 | -1.17 | 0.261 | 0.462 | 1.000 | 3832 | tags=41%, list=30%, signal=58% |
| 209 | GOMF\_PROTEIN\_TRANSMEMBRANE\_TRANSPORTER\_ACTIVITY |  | 18 | -0.39 | -1.16 | 0.263 | 0.471 | 1.000 | 3028 | tags=33%, list=23%, signal=43% |
| 210 | GOMF\_LAMININ\_BINDING |  | 23 | -0.37 | -1.16 | 0.256 | 0.474 | 1.000 | 4036 | tags=52%, list=31%, signal=76% |
| 211 | GOMF\_TRANSCRIPTION\_COREPRESSOR\_BINDING |  | 38 | -0.33 | -1.16 | 0.247 | 0.473 | 1.000 | 3296 | tags=32%, list=26%, signal=42% |
| 212 | GOMF\_IONOTROPIC\_GLUTAMATE\_RECEPTOR\_ACTIVITY |  | 16 | -0.41 | -1.16 | 0.282 | 0.473 | 1.000 | 1596 | tags=31%, list=12%, signal=36% |
| 213 | GOMF\_STRUCTURAL\_CONSTITUENT\_OF\_SYNAPSE |  | 18 | -0.40 | -1.16 | 0.259 | 0.471 | 1.000 | 1825 | tags=22%, list=14%, signal=26% |
| 214 | GOMF\_PHOSPHATIDYLINOSITOL\_3\_PHOSPHATE\_BINDING |  | 29 | -0.35 | -1.16 | 0.250 | 0.469 | 1.000 | 413 | tags=10%, list=3%, signal=11% |
| 215 | GOMF\_ESTROGEN\_RECEPTOR\_BINDING |  | 33 | -0.34 | -1.16 | 0.237 | 0.473 | 1.000 | 1756 | tags=21%, list=14%, signal=24% |
| 216 | GOMF\_DOUBLE\_STRANDED\_RNA\_BINDING |  | 68 | -0.29 | -1.16 | 0.218 | 0.476 | 1.000 | 2842 | tags=32%, list=22%, signal=41% |
| 217 | GOMF\_NAD\_DEPENDENT\_PROTEIN\_DEACETYLASE\_ACTIVITY |  | 16 | -0.39 | -1.15 | 0.267 | 0.488 | 1.000 | 301 | tags=13%, list=2%, signal=13% |
| 218 | GOMF\_SYMPORTER\_ACTIVITY |  | 101 | -0.26 | -1.14 | 0.207 | 0.501 | 1.000 | 4470 | tags=45%, list=35%, signal=68% |
| 219 | GOMF\_ATPASE\_COUPLED\_INORGANIC\_ANION\_TRANSMEMBRANE\_TRANSPORTER\_ACTIVITY |  | 21 | -0.37 | -1.14 | 0.279 | 0.508 | 1.000 | 4107 | tags=48%, list=32%, signal=70% |
| 220 | GOMF\_RNA\_POLYMERASE\_II\_CORE\_PROMOTER\_SEQUENCE\_SPECIFIC\_DNA\_BINDING |  | 20 | -0.37 | -1.14 | 0.297 | 0.507 | 1.000 | 2262 | tags=30%, list=18%, signal=36% |
| 221 | GOMF\_HORMONE\_RECEPTOR\_BINDING |  | 26 | -0.34 | -1.14 | 0.289 | 0.513 | 1.000 | 4162 | tags=46%, list=32%, signal=68% |
| 222 | GOMF\_SPECTRIN\_BINDING |  | 23 | -0.35 | -1.13 | 0.276 | 0.517 | 1.000 | 4077 | tags=43%, list=32%, signal=63% |
| 223 | GOMF\_ATP\_DEPENDENT\_ACTIVITY |  | 435 | -0.22 | -1.13 | 0.153 | 0.515 | 1.000 | 3119 | tags=27%, list=24%, signal=35% |
| 224 | GOMF\_SEMAPHORIN\_RECEPTOR\_BINDING |  | 21 | -0.37 | -1.13 | 0.280 | 0.518 | 1.000 | 2886 | tags=43%, list=22%, signal=55% |
| 225 | GOMF\_SIGNALING\_RECEPTOR\_REGULATOR\_ACTIVITY |  | 409 | -0.22 | -1.13 | 0.154 | 0.521 | 1.000 | 2486 | tags=22%, list=19%, signal=26% |
| 226 | GOMF\_DNA\_POLYMERASE\_BINDING |  | 18 | -0.38 | -1.13 | 0.306 | 0.520 | 1.000 | 2893 | tags=33%, list=22%, signal=43% |
| 227 | GOMF\_AMIDE\_BINDING |  | 344 | -0.22 | -1.13 | 0.155 | 0.519 | 1.000 | 2176 | tags=20%, list=17%, signal=23% |
| 228 | GOMF\_ENZYME\_INHIBITOR\_ACTIVITY |  | 288 | -0.22 | -1.12 | 0.177 | 0.521 | 1.000 | 3065 | tags=28%, list=24%, signal=36% |
| 229 | GOMF\_S\_ACYLTRANSFERASE\_ACTIVITY |  | 16 | -0.39 | -1.12 | 0.316 | 0.525 | 1.000 | 2268 | tags=44%, list=18%, signal=53% |
| 230 | GOMF\_SULFUR\_COMPOUND\_TRANSMEMBRANE\_TRANSPORTER\_ACTIVITY |  | 41 | -0.30 | -1.12 | 0.276 | 0.525 | 1.000 | 4049 | tags=46%, list=31%, signal=67% |
| 231 | GOMF\_INSULIN\_LIKE\_GROWTH\_FACTOR\_BINDING |  | 26 | -0.34 | -1.12 | 0.302 | 0.528 | 1.000 | 656 | tags=15%, list=5%, signal=16% |
| 232 | GOMF\_RRNA\_BINDING |  | 55 | -0.28 | -1.12 | 0.264 | 0.528 | 1.000 | 2701 | tags=33%, list=21%, signal=41% |
| 233 | GOMF\_ENDOPEPTIDASE\_ACTIVITY |  | 315 | -0.22 | -1.12 | 0.169 | 0.529 | 1.000 | 3221 | tags=29%, list=25%, signal=38% |
| 234 | GOMF\_LIGAND\_GATED\_CATION\_CHANNEL\_ACTIVITY |  | 97 | -0.26 | -1.12 | 0.245 | 0.530 | 1.000 | 4259 | tags=41%, list=33%, signal=61% |
| 235 | GOMF\_OMEGA\_PEPTIDASE\_ACTIVITY |  | 72 | -0.27 | -1.11 | 0.260 | 0.532 | 1.000 | 1847 | tags=22%, list=14%, signal=26% |
| 236 | GOMF\_UBIQUITIN\_LIKE\_PROTEIN\_LIGASE\_BINDING |  | 280 | -0.22 | -1.11 | 0.219 | 0.530 | 1.000 | 2885 | tags=25%, list=22%, signal=32% |
| 237 | GOMF\_2\_OXOGLUTARATE\_DEPENDENT\_DIOXYGENASE\_ACTIVITY |  | 46 | -0.30 | -1.11 | 0.290 | 0.530 | 1.000 | 2940 | tags=35%, list=23%, signal=45% |
| 238 | GOMF\_CATALYTIC\_ACTIVITY\_ACTING\_ON\_DNA |  | 184 | -0.23 | -1.11 | 0.217 | 0.528 | 1.000 | 2893 | tags=29%, list=22%, signal=37% |
| 239 | GOMF\_SODIUM\_CHANNEL\_ACTIVITY |  | 36 | -0.31 | -1.11 | 0.308 | 0.526 | 1.000 | 5417 | tags=53%, list=42%, signal=91% |
| 240 | GOMF\_PHOSPHATASE\_BINDING |  | 157 | -0.24 | -1.11 | 0.245 | 0.530 | 1.000 | 2233 | tags=24%, list=17%, signal=28% |
| 241 | GOMF\_CHEMOKINE\_RECEPTOR\_BINDING |  | 54 | -0.29 | -1.10 | 0.294 | 0.542 | 1.000 | 3548 | tags=37%, list=28%, signal=51% |
| 242 | GOMF\_CALCIUM\_ION\_TRANSMEMBRANE\_TRANSPORTER\_ACTIVITY |  | 105 | -0.25 | -1.10 | 0.267 | 0.541 | 1.000 | 3081 | tags=30%, list=24%, signal=38% |
| 243 | GOMF\_PROTEIN\_DEACETYLASE\_ACTIVITY |  | 18 | -0.37 | -1.10 | 0.314 | 0.541 | 1.000 | 5069 | tags=61%, list=39%, signal=101% |
| 244 | GOMF\_N\_ACYLTRANSFERASE\_ACTIVITY |  | 65 | -0.27 | -1.10 | 0.294 | 0.546 | 1.000 | 1362 | tags=17%, list=11%, signal=19% |
| 245 | GOMF\_SH3\_DOMAIN\_BINDING |  | 99 | -0.25 | -1.09 | 0.281 | 0.562 | 1.000 | 1357 | tags=14%, list=11%, signal=16% |
| 246 | GOMF\_TRANSITION\_METAL\_ION\_TRANSMEMBRANE\_TRANSPORTER\_ACTIVITY |  | 26 | -0.33 | -1.09 | 0.322 | 0.572 | 1.000 | 2963 | tags=38%, list=23%, signal=50% |
| 247 | GOMF\_PENTOSYLTRANSFERASE\_ACTIVITY |  | 40 | -0.30 | -1.08 | 0.328 | 0.594 | 1.000 | 2136 | tags=23%, list=17%, signal=27% |
| 248 | GOMF\_PEPTIDE\_HORMONE\_RECEPTOR\_BINDING |  | 15 | -0.38 | -1.07 | 0.367 | 0.614 | 1.000 | 4162 | tags=53%, list=32%, signal=79% |
| 249 | GOMF\_SIGNALING\_ADAPTOR\_ACTIVITY |  | 67 | -0.27 | -1.07 | 0.339 | 0.614 | 1.000 | 3430 | tags=34%, list=27%, signal=47% |
| 250 | GOMF\_CALMODULIN\_DEPENDENT\_PROTEIN\_KINASE\_ACTIVITY |  | 22 | -0.34 | -1.07 | 0.360 | 0.613 | 1.000 | 2452 | tags=27%, list=19%, signal=34% |
| 251 | GOMF\_BASAL\_TRANSCRIPTION\_MACHINERY\_BINDING |  | 49 | -0.28 | -1.07 | 0.324 | 0.611 | 1.000 | 3055 | tags=35%, list=24%, signal=45% |
| 252 | GOMF\_PHOSPHOTYROSINE\_RESIDUE\_BINDING |  | 39 | -0.29 | -1.07 | 0.354 | 0.610 | 1.000 | 1946 | tags=26%, list=15%, signal=30% |
| 253 | GOMF\_PHOSPHATIDYLCHOLINE\_TRANSPORTER\_ACTIVITY |  | 17 | -0.36 | -1.07 | 0.380 | 0.613 | 1.000 | 4046 | tags=53%, list=31%, signal=77% |
| 254 | GOMF\_NADPLUS\_ADP\_RIBOSYLTRANSFERASE\_ACTIVITY |  | 22 | -0.34 | -1.07 | 0.373 | 0.613 | 1.000 | 2136 | tags=23%, list=17%, signal=27% |
| 255 | GOMF\_FLIPPASE\_ACTIVITY |  | 15 | -0.38 | -1.06 | 0.383 | 0.613 | 1.000 | 5143 | tags=67%, list=40%, signal=111% |
| 256 | GOMF\_PROTEIN\_PHOSPHATASE\_BINDING |  | 125 | -0.23 | -1.06 | 0.350 | 0.621 | 1.000 | 2151 | tags=22%, list=17%, signal=26% |
| 257 | GOMF\_SERINE\_HYDROLASE\_ACTIVITY |  | 148 | -0.23 | -1.06 | 0.333 | 0.619 | 1.000 | 3210 | tags=29%, list=25%, signal=38% |
| 258 | GOMF\_RNA\_POLYMERASE\_II\_GENERAL\_TRANSCRIPTION\_INITIATION\_FACTOR\_ACTIVITY |  | 33 | -0.30 | -1.06 | 0.360 | 0.620 | 1.000 | 1764 | tags=21%, list=14%, signal=25% |
| 259 | GOMF\_RNA\_POLYMERASE\_CORE\_ENZYME\_BINDING |  | 31 | -0.31 | -1.06 | 0.375 | 0.623 | 1.000 | 3055 | tags=39%, list=24%, signal=51% |
| 260 | GOMF\_VOLTAGE\_GATED\_POTASSIUM\_CHANNEL\_ACTIVITY |  | 64 | -0.26 | -1.06 | 0.354 | 0.624 | 1.000 | 2925 | tags=30%, list=23%, signal=38% |
| 261 | GOMF\_RETINOIC\_ACID\_RECEPTOR\_BINDING |  | 24 | -0.32 | -1.06 | 0.401 | 0.623 | 1.000 | 4421 | tags=46%, list=34%, signal=70% |
| 262 | GOMF\_CHEMOREPELLENT\_ACTIVITY |  | 25 | -0.32 | -1.06 | 0.381 | 0.621 | 1.000 | 1776 | tags=28%, list=14%, signal=32% |
| 263 | GOMF\_CHAPERONE\_BINDING |  | 90 | -0.25 | -1.05 | 0.360 | 0.622 | 1.000 | 2861 | tags=28%, list=22%, signal=35% |
| 264 | GOMF\_PHOSPHATIDYLINOSITOL\_4\_5\_BISPHOSPHATE\_BINDING |  | 61 | -0.26 | -1.05 | 0.364 | 0.621 | 1.000 | 2327 | tags=21%, list=18%, signal=26% |
| 265 | GOMF\_TRANSLATION\_REGULATOR\_ACTIVITY\_NUCLEIC\_ACID\_BINDING |  | 83 | -0.25 | -1.05 | 0.357 | 0.620 | 1.000 | 3193 | tags=34%, list=25%, signal=45% |
| 266 | GOMF\_FIBRONECTIN\_BINDING |  | 25 | -0.33 | -1.05 | 0.394 | 0.636 | 1.000 | 2592 | tags=32%, list=20%, signal=40% |
| 267 | GOMF\_CALMODULIN\_BINDING |  | 172 | -0.22 | -1.04 | 0.341 | 0.644 | 1.000 | 2141 | tags=19%, list=17%, signal=22% |
| 268 | GOMF\_FOUR\_WAY\_JUNCTION\_DNA\_BINDING |  | 15 | -0.37 | -1.04 | 0.394 | 0.643 | 1.000 | 732 | tags=20%, list=6%, signal=21% |
| 269 | GOMF\_UBIQUITIN\_LIKE\_PROTEIN\_SPECIFIC\_PROTEASE\_ACTIVITY |  | 82 | -0.25 | -1.04 | 0.361 | 0.643 | 1.000 | 1877 | tags=23%, list=15%, signal=27% |
| 270 | GOMF\_MICROTUBULE\_MOTOR\_ACTIVITY |  | 47 | -0.27 | -1.04 | 0.398 | 0.650 | 1.000 | 3008 | tags=23%, list=23%, signal=30% |
| 271 | GOMF\_UNFOLDED\_PROTEIN\_BINDING |  | 97 | -0.24 | -1.03 | 0.384 | 0.659 | 1.000 | 3876 | tags=36%, list=30%, signal=51% |
| 272 | GOMF\_MITOGEN\_ACTIVATED\_PROTEIN\_KINASE\_BINDING |  | 26 | -0.32 | -1.03 | 0.411 | 0.659 | 1.000 | 3955 | tags=35%, list=31%, signal=50% |
| 273 | GOMF\_PROTEIN\_HETERODIMERIZATION\_ACTIVITY |  | 263 | -0.21 | -1.03 | 0.391 | 0.661 | 1.000 | 1961 | tags=17%, list=15%, signal=19% |
| 274 | GOMF\_CYTOKINE\_RECEPTOR\_BINDING |  | 226 | -0.21 | -1.03 | 0.376 | 0.664 | 1.000 | 3553 | tags=28%, list=28%, signal=38% |
| 275 | GOMF\_NEUROTRANSMITTER\_RECEPTOR\_ACTIVITY |  | 91 | -0.24 | -1.03 | 0.404 | 0.665 | 1.000 | 3003 | tags=30%, list=23%, signal=38% |
| 276 | GOMF\_CALCIUM\_CHANNEL\_REGULATOR\_ACTIVITY |  | 36 | -0.29 | -1.03 | 0.425 | 0.667 | 1.000 | 4607 | tags=50%, list=36%, signal=78% |
| 277 | GOMF\_TETRAPYRROLE\_BINDING |  | 122 | -0.23 | -1.02 | 0.398 | 0.673 | 1.000 | 3720 | tags=34%, list=29%, signal=47% |
| 278 | GOMF\_PROTEIN\_C\_TERMINUS\_BINDING |  | 166 | -0.22 | -1.02 | 0.409 | 0.675 | 1.000 | 2134 | tags=20%, list=17%, signal=24% |
| 279 | GOMF\_EXTRACELLULAR\_MATRIX\_STRUCTURAL\_CONSTITUENT |  | 143 | -0.22 | -1.02 | 0.425 | 0.674 | 1.000 | 4118 | tags=38%, list=32%, signal=55% |
| 280 | GOMF\_INSULIN\_LIKE\_GROWTH\_FACTOR\_RECEPTOR\_BINDING |  | 15 | -0.36 | -1.02 | 0.417 | 0.673 | 1.000 | 2013 | tags=33%, list=16%, signal=39% |
| 281 | GOMF\_CELL\_ADHESION\_MEDIATOR\_ACTIVITY |  | 49 | -0.27 | -1.02 | 0.417 | 0.680 | 1.000 | 4377 | tags=47%, list=34%, signal=71% |
| 282 | GOMF\_CYTOKINE\_ACTIVITY |  | 182 | -0.21 | -1.01 | 0.410 | 0.689 | 1.000 | 3413 | tags=28%, list=26%, signal=38% |
| 283 | GOMF\_LIPID\_TRANSPORTER\_ACTIVITY |  | 120 | -0.22 | -1.01 | 0.415 | 0.688 | 1.000 | 4046 | tags=34%, list=31%, signal=49% |
| 284 | GOMF\_GTPASE\_ACTIVITY |  | 248 | -0.20 | -1.01 | 0.424 | 0.686 | 1.000 | 1941 | tags=17%, list=15%, signal=20% |
| 285 | GOMF\_CYTOSKELETAL\_ANCHOR\_ACTIVITY |  | 19 | -0.33 | -1.01 | 0.434 | 0.691 | 1.000 | 3147 | tags=42%, list=24%, signal=56% |
| 286 | GOMF\_VOLTAGE\_GATED\_CATION\_CHANNEL\_ACTIVITY |  | 101 | -0.23 | -1.00 | 0.461 | 0.705 | 1.000 | 3035 | tags=28%, list=24%, signal=36% |
| 287 | GOMF\_G\_PROTEIN\_BETA\_GAMMA\_SUBUNIT\_COMPLEX\_BINDING |  | 22 | -0.32 | -1.00 | 0.438 | 0.715 | 1.000 | 887 | tags=14%, list=7%, signal=15% |
| 288 | GOMF\_TRANSMEMBRANE\_RECEPTOR\_PROTEIN\_SERINE\_THREONINE\_KINASE\_ACTIVITY |  | 16 | -0.34 | -1.00 | 0.485 | 0.716 | 1.000 | 2331 | tags=25%, list=18%, signal=30% |
| 289 | GOMF\_ACYLGLYCEROL\_O\_ACYLTRANSFERASE\_ACTIVITY |  | 17 | -0.34 | -1.00 | 0.458 | 0.713 | 1.000 | 4468 | tags=53%, list=35%, signal=81% |
| 290 | GOMF\_CCR\_CHEMOKINE\_RECEPTOR\_BINDING |  | 33 | -0.29 | -0.99 | 0.478 | 0.717 | 1.000 | 4663 | tags=48%, list=36%, signal=76% |
| 291 | GOMF\_CATION\_CHLORIDE\_SYMPORTER\_ACTIVITY |  | 15 | -0.35 | -0.99 | 0.455 | 0.714 | 1.000 | 4009 | tags=47%, list=31%, signal=68% |
| 292 | GOMF\_MHC\_CLASS\_II\_PROTEIN\_COMPLEX\_BINDING |  | 24 | -0.31 | -0.99 | 0.502 | 0.721 | 1.000 | 765 | tags=17%, list=6%, signal=18% |
| 293 | GOMF\_ANDROGEN\_RECEPTOR\_BINDING |  | 26 | -0.30 | -0.99 | 0.480 | 0.722 | 1.000 | 1574 | tags=23%, list=12%, signal=26% |
| 294 | GOMF\_MOLECULAR\_FUNCTION\_INHIBITOR\_ACTIVITY |  | 43 | -0.27 | -0.99 | 0.480 | 0.721 | 1.000 | 3419 | tags=33%, list=27%, signal=44% |
| 295 | GOMF\_INSULIN\_RECEPTOR\_BINDING |  | 22 | -0.31 | -0.98 | 0.455 | 0.738 | 1.000 | 2187 | tags=32%, list=17%, signal=38% |
| 296 | GOMF\_LIGAND\_GATED\_ION\_CHANNEL\_ACTIVITY |  | 122 | -0.22 | -0.98 | 0.509 | 0.753 | 1.000 | 4259 | tags=39%, list=33%, signal=57% |
| 297 | GOMF\_PROTEIN\_TYROSINE\_KINASE\_BINDING |  | 91 | -0.22 | -0.98 | 0.519 | 0.751 | 1.000 | 2624 | tags=26%, list=20%, signal=33% |
| 298 | GOMF\_PROTEIN\_N\_TERMINUS\_BINDING |  | 97 | -0.23 | -0.97 | 0.529 | 0.752 | 1.000 | 2893 | tags=24%, list=22%, signal=30% |
| 299 | GOMF\_PEPTIDE\_ANTIGEN\_BINDING |  | 24 | -0.30 | -0.97 | 0.506 | 0.766 | 1.000 | 1286 | tags=21%, list=10%, signal=23% |
| 300 | GOMF\_METALLOPEPTIDASE\_ACTIVITY |  | 152 | -0.21 | -0.97 | 0.536 | 0.766 | 1.000 | 4451 | tags=41%, list=35%, signal=63% |
| 301 | GOMF\_PHOSPHATASE\_INHIBITOR\_ACTIVITY |  | 28 | -0.29 | -0.97 | 0.520 | 0.767 | 1.000 | 3394 | tags=39%, list=26%, signal=53% |
| 302 | GOMF\_UBIQUITIN\_LIKE\_PROTEIN\_BINDING |  | 99 | -0.22 | -0.96 | 0.544 | 0.779 | 1.000 | 2677 | tags=25%, list=21%, signal=32% |
| 303 | GOMF\_PHOSPHORIC\_DIESTER\_HYDROLASE\_ACTIVITY |  | 72 | -0.23 | -0.96 | 0.513 | 0.778 | 1.000 | 2461 | tags=25%, list=19%, signal=31% |
| 304 | GOMF\_BHLH\_TRANSCRIPTION\_FACTOR\_BINDING |  | 28 | -0.28 | -0.96 | 0.506 | 0.776 | 1.000 | 2110 | tags=25%, list=16%, signal=30% |
| 305 | GOMF\_CADHERIN\_BINDING\_INVOLVED\_IN\_CELL\_CELL\_ADHESION |  | 16 | -0.33 | -0.96 | 0.515 | 0.777 | 1.000 | 4308 | tags=50%, list=33%, signal=75% |
| 306 | GOMF\_NON\_MEMBRANE\_SPANNING\_PROTEIN\_TYROSINE\_KINASE\_ACTIVITY |  | 43 | -0.26 | -0.96 | 0.538 | 0.781 | 1.000 | 4063 | tags=35%, list=31%, signal=51% |
| 307 | GOMF\_UBIQUITIN\_LIKE\_PROTEIN\_CONJUGATING\_ENZYME\_BINDING |  | 28 | -0.28 | -0.95 | 0.524 | 0.805 | 1.000 | 2542 | tags=32%, list=20%, signal=40% |
| 308 | GOMF\_NUCLEOTIDE\_RECEPTOR\_ACTIVITY |  | 18 | -0.32 | -0.95 | 0.515 | 0.805 | 1.000 | 3935 | tags=39%, list=31%, signal=56% |
| 309 | GOMF\_PROLINE\_RICH\_REGION\_BINDING |  | 16 | -0.32 | -0.94 | 0.558 | 0.813 | 1.000 | 1533 | tags=19%, list=12%, signal=21% |
| 310 | GOMF\_AMYLOID\_BETA\_BINDING |  | 82 | -0.23 | -0.94 | 0.577 | 0.813 | 1.000 | 1424 | tags=15%, list=11%, signal=16% |
| 311 | GOMF\_NEUROTRANSMITTER\_TRANSMEMBRANE\_TRANSPORTER\_ACTIVITY |  | 16 | -0.32 | -0.93 | 0.553 | 0.826 | 1.000 | 3562 | tags=31%, list=28%, signal=43% |
| 312 | GOMF\_THYROID\_HORMONE\_RECEPTOR\_BINDING |  | 28 | -0.28 | -0.93 | 0.555 | 0.827 | 1.000 | 1756 | tags=21%, list=14%, signal=25% |
| 313 | GOMF\_TRANSLATION\_INITIATION\_FACTOR\_ACTIVITY |  | 44 | -0.25 | -0.93 | 0.582 | 0.833 | 1.000 | 3651 | tags=36%, list=28%, signal=51% |
| 314 | GOMF\_MACROMOLECULE\_TRANSMEMBRANE\_TRANSPORTER\_ACTIVITY |  | 22 | -0.29 | -0.93 | 0.567 | 0.839 | 1.000 | 3028 | tags=27%, list=23%, signal=36% |
| 315 | GOMF\_IRON\_ION\_BINDING |  | 119 | -0.21 | -0.93 | 0.603 | 0.837 | 1.000 | 4113 | tags=39%, list=32%, signal=57% |
| 316 | GOMF\_CHANNEL\_INHIBITOR\_ACTIVITY |  | 25 | -0.29 | -0.93 | 0.549 | 0.836 | 1.000 | 4358 | tags=44%, list=34%, signal=66% |
| 317 | GOMF\_PEPTIDASE\_ACTIVITY |  | 449 | -0.18 | -0.92 | 0.709 | 0.836 | 1.000 | 3634 | tags=31%, list=28%, signal=41% |
| 318 | GOMF\_MOLECULAR\_FUNCTION\_ACTIVATOR\_ACTIVITY |  | 28 | -0.28 | -0.92 | 0.576 | 0.835 | 1.000 | 3769 | tags=43%, list=29%, signal=60% |
| 319 | GOMF\_TRANSCRIPTION\_REGULATOR\_INHIBITOR\_ACTIVITY |  | 15 | -0.32 | -0.92 | 0.541 | 0.835 | 1.000 | 1554 | tags=20%, list=12%, signal=23% |
| 320 | GOMF\_STEROID\_HORMONE\_RECEPTOR\_ACTIVITY |  | 21 | -0.30 | -0.92 | 0.570 | 0.832 | 1.000 | 1792 | tags=24%, list=14%, signal=28% |
| 321 | GOMF\_LIGASE\_ACTIVITY\_FORMING\_CARBON\_NITROGEN\_BONDS |  | 33 | -0.26 | -0.92 | 0.596 | 0.846 | 1.000 | 2653 | tags=30%, list=21%, signal=38% |
| 322 | GOMF\_MOLECULAR\_ADAPTOR\_ACTIVITY |  | 307 | -0.18 | -0.92 | 0.710 | 0.845 | 1.000 | 3168 | tags=26%, list=25%, signal=34% |
| 323 | GOMF\_RECEPTOR\_TYROSINE\_KINASE\_BINDING |  | 66 | -0.22 | -0.91 | 0.623 | 0.849 | 1.000 | 2624 | tags=27%, list=20%, signal=34% |
| 324 | GOMF\_SERINE\_TYPE\_ENDOPEPTIDASE\_INHIBITOR\_ACTIVITY |  | 64 | -0.23 | -0.91 | 0.628 | 0.848 | 1.000 | 3065 | tags=33%, list=24%, signal=43% |
| 325 | GOMF\_ATPASE\_COUPLED\_INTRAMEMBRANE\_LIPID\_TRANSPORTER\_ACTIVITY |  | 24 | -0.28 | -0.91 | 0.585 | 0.846 | 1.000 | 4046 | tags=42%, list=31%, signal=61% |
| 326 | GOMF\_PROTEIN\_MACROMOLECULE\_ADAPTOR\_ACTIVITY |  | 256 | -0.18 | -0.91 | 0.718 | 0.850 | 1.000 | 3168 | tags=27%, list=25%, signal=35% |
| 327 | GOMF\_ACTIN\_FILAMENT\_BINDING |  | 178 | -0.19 | -0.91 | 0.685 | 0.851 | 1.000 | 4746 | tags=38%, list=37%, signal=60% |
| 328 | GOMF\_TRANSLATION\_REPRESSOR\_ACTIVITY |  | 18 | -0.30 | -0.90 | 0.613 | 0.869 | 1.000 | 4965 | tags=50%, list=38%, signal=81% |
| 329 | GOMF\_TUMOR\_NECROSIS\_FACTOR\_RECEPTOR\_SUPERFAMILY\_BINDING |  | 45 | -0.24 | -0.90 | 0.632 | 0.869 | 1.000 | 1005 | tags=13%, list=8%, signal=14% |
| 330 | GOMF\_PLUS\_END\_DIRECTED\_MICROTUBULE\_MOTOR\_ACTIVITY |  | 16 | -0.31 | -0.90 | 0.602 | 0.870 | 1.000 | 2960 | tags=25%, list=23%, signal=32% |
| 331 | GOMF\_VOLTAGE\_GATED\_SODIUM\_CHANNEL\_ACTIVITY |  | 19 | -0.30 | -0.90 | 0.618 | 0.869 | 1.000 | 5417 | tags=53%, list=42%, signal=91% |
| 332 | GOMF\_FIBROBLAST\_GROWTH\_FACTOR\_BINDING |  | 20 | -0.29 | -0.90 | 0.603 | 0.867 | 1.000 | 2592 | tags=25%, list=20%, signal=31% |
| 333 | GOMF\_CYSTEINE\_TYPE\_ENDOPEPTIDASE\_REGULATOR\_ACTIVITY\_INVOLVED\_IN\_APOPTOTIC\_PROCESS |  | 36 | -0.25 | -0.90 | 0.629 | 0.864 | 1.000 | 2389 | tags=25%, list=19%, signal=31% |
| 334 | GOMF\_CYSTEINE\_TYPE\_ENDOPEPTIDASE\_ACTIVITY |  | 80 | -0.21 | -0.89 | 0.685 | 0.873 | 1.000 | 2659 | tags=26%, list=21%, signal=33% |
| 335 | GOMF\_VOLTAGE\_GATED\_CHANNEL\_ACTIVITY |  | 143 | -0.19 | -0.89 | 0.715 | 0.872 | 1.000 | 4412 | tags=38%, list=34%, signal=57% |
| 336 | GOMF\_CATION\_TRANSMEMBRANE\_TRANSPORTER\_ACTIVITY |  | 471 | -0.17 | -0.89 | 0.824 | 0.870 | 1.000 | 3035 | tags=25%, list=24%, signal=32% |
| 337 | GOMF\_GTP\_DEPENDENT\_PROTEIN\_BINDING |  | 19 | -0.30 | -0.89 | 0.611 | 0.869 | 1.000 | 4403 | tags=58%, list=34%, signal=88% |
| 338 | GOMF\_CELL\_CELL\_ADHESION\_MEDIATOR\_ACTIVITY |  | 40 | -0.25 | -0.89 | 0.625 | 0.873 | 1.000 | 4377 | tags=43%, list=34%, signal=64% |
| 339 | GOMF\_EXTRACELLULAR\_LIGAND\_GATED\_ION\_CHANNEL\_ACTIVITY |  | 63 | -0.22 | -0.88 | 0.664 | 0.884 | 1.000 | 3003 | tags=29%, list=23%, signal=37% |
| 340 | GOMF\_LYSOPHOSPHOLIPID\_ACYLTRANSFERASE\_ACTIVITY |  | 16 | -0.30 | -0.88 | 0.620 | 0.887 | 1.000 | 4468 | tags=50%, list=35%, signal=76% |
| 341 | GOMF\_MHC\_PROTEIN\_COMPLEX\_BINDING |  | 32 | -0.25 | -0.87 | 0.676 | 0.903 | 1.000 | 765 | tags=13%, list=6%, signal=13% |
| 342 | GOMF\_PROTEIN\_KINASE\_A\_REGULATORY\_SUBUNIT\_BINDING |  | 21 | -0.28 | -0.87 | 0.652 | 0.908 | 1.000 | 3117 | tags=43%, list=24%, signal=56% |
| 343 | GOMF\_ORGANOPHOSPHATE\_ESTER\_TRANSMEMBRANE\_TRANSPORTER\_ACTIVITY |  | 19 | -0.29 | -0.87 | 0.640 | 0.915 | 1.000 | 4049 | tags=42%, list=31%, signal=61% |
| 344 | GOMF\_MICROTUBULE\_PLUS\_END\_BINDING |  | 20 | -0.28 | -0.86 | 0.680 | 0.914 | 1.000 | 2462 | tags=25%, list=19%, signal=31% |
| 345 | GOMF\_RECEPTOR\_SERINE\_THREONINE\_KINASE\_BINDING |  | 23 | -0.27 | -0.86 | 0.674 | 0.916 | 1.000 | 5124 | tags=61%, list=40%, signal=101% |
| 346 | GOMF\_ACYLTRANSFERASE\_ACTIVITY |  | 166 | -0.19 | -0.86 | 0.807 | 0.918 | 1.000 | 2864 | tags=23%, list=22%, signal=30% |
| 347 | GOMF\_SPHINGOLIPID\_BINDING |  | 21 | -0.28 | -0.86 | 0.672 | 0.922 | 1.000 | 439 | tags=10%, list=3%, signal=10% |
| 348 | GOMF\_PHOSPHOLIPID\_BINDING |  | 357 | -0.17 | -0.86 | 0.867 | 0.924 | 1.000 | 2456 | tags=19%, list=19%, signal=23% |
| 349 | GOMF\_DNA\_SECONDARY\_STRUCTURE\_BINDING |  | 31 | -0.25 | -0.85 | 0.699 | 0.925 | 1.000 | 975 | tags=13%, list=8%, signal=14% |
| 350 | GOMF\_MOLECULAR\_CARRIER\_ACTIVITY |  | 61 | -0.21 | -0.85 | 0.719 | 0.929 | 1.000 | 2543 | tags=26%, list=20%, signal=33% |
| 351 | GOMF\_SOLUTE\_CATION\_ANTIPORTER\_ACTIVITY |  | 23 | -0.27 | -0.85 | 0.717 | 0.931 | 1.000 | 710 | tags=17%, list=6%, signal=18% |
| 352 | GOMF\_INTRAMOLECULAR\_TRANSFERASE\_ACTIVITY |  | 18 | -0.29 | -0.85 | 0.677 | 0.933 | 1.000 | 3494 | tags=39%, list=27%, signal=53% |
| 353 | GOMF\_DIOXYGENASE\_ACTIVITY |  | 73 | -0.21 | -0.85 | 0.761 | 0.933 | 1.000 | 1508 | tags=18%, list=12%, signal=20% |
| 354 | GOMF\_GROWTH\_FACTOR\_ACTIVITY |  | 144 | -0.18 | -0.84 | 0.822 | 0.939 | 1.000 | 3374 | tags=26%, list=26%, signal=35% |
| 355 | GOMF\_PHOSPHATASE\_ACTIVATOR\_ACTIVITY |  | 22 | -0.27 | -0.84 | 0.710 | 0.937 | 1.000 | 3348 | tags=36%, list=26%, signal=49% |
| 356 | GOMF\_CLATHRIN\_BINDING |  | 52 | -0.22 | -0.84 | 0.748 | 0.935 | 1.000 | 1185 | tags=13%, list=9%, signal=15% |
| 357 | GOMF\_CATION\_CHANNEL\_ACTIVITY |  | 243 | -0.17 | -0.83 | 0.896 | 0.947 | 1.000 | 3081 | tags=24%, list=24%, signal=31% |
| 358 | GOMF\_PROTEIN\_FOLDING\_CHAPERONE |  | 37 | -0.23 | -0.83 | 0.746 | 0.951 | 1.000 | 3974 | tags=41%, list=31%, signal=58% |
| 359 | GOMF\_MICROTUBULE\_BINDING |  | 198 | -0.17 | -0.83 | 0.857 | 0.953 | 1.000 | 2873 | tags=21%, list=22%, signal=26% |
| 360 | GOMF\_RNA\_POLYMERASE\_II\_GENERAL\_TRANSCRIPTION\_INITIATION\_FACTOR\_BINDING |  | 23 | -0.26 | -0.83 | 0.720 | 0.951 | 1.000 | 3055 | tags=35%, list=24%, signal=45% |
| 361 | GOMF\_MHC\_CLASS\_I\_PROTEIN\_BINDING |  | 20 | -0.27 | -0.83 | 0.728 | 0.951 | 1.000 | 1286 | tags=15%, list=10%, signal=17% |
| 362 | GOMF\_WW\_DOMAIN\_BINDING |  | 23 | -0.26 | -0.83 | 0.727 | 0.949 | 1.000 | 3957 | tags=43%, list=31%, signal=63% |
| 363 | GOMF\_TRANSMITTER\_GATED\_CHANNEL\_ACTIVITY |  | 53 | -0.22 | -0.82 | 0.763 | 0.952 | 1.000 | 4259 | tags=42%, list=33%, signal=62% |
| 364 | GOMF\_XENOBIOTIC\_TRANSMEMBRANE\_TRANSPORTER\_ACTIVITY |  | 25 | -0.25 | -0.82 | 0.710 | 0.950 | 1.000 | 5534 | tags=64%, list=43%, signal=112% |
| 365 | GOMF\_INTRAMEMBRANE\_LIPID\_TRANSPORTER\_ACTIVITY |  | 33 | -0.24 | -0.82 | 0.730 | 0.950 | 1.000 | 4046 | tags=36%, list=31%, signal=53% |
| 366 | GOMF\_GATED\_CHANNEL\_ACTIVITY |  | 253 | -0.16 | -0.82 | 0.919 | 0.950 | 1.000 | 4412 | tags=36%, list=34%, signal=54% |
| 367 | GOMF\_ACTIN\_BINDING |  | 355 | -0.16 | -0.81 | 0.954 | 0.958 | 1.000 | 2935 | tags=19%, list=23%, signal=24% |
| 368 | GOMF\_GAMMA\_TUBULIN\_BINDING |  | 24 | -0.25 | -0.81 | 0.784 | 0.957 | 1.000 | 4214 | tags=42%, list=33%, signal=62% |
| 369 | GOMF\_DEOXYRIBONUCLEASE\_ACTIVITY |  | 49 | -0.22 | -0.81 | 0.802 | 0.960 | 1.000 | 1955 | tags=20%, list=15%, signal=24% |
| 370 | GOMF\_OPSONIN\_BINDING |  | 15 | -0.28 | -0.81 | 0.729 | 0.960 | 1.000 | 2959 | tags=33%, list=23%, signal=43% |
| 371 | GOMF\_EXORIBONUCLEASE\_ACTIVITY\_PRODUCING\_5\_PHOSPHOMONOESTERS |  | 27 | -0.24 | -0.81 | 0.761 | 0.963 | 1.000 | 2498 | tags=26%, list=19%, signal=32% |
| 372 | GOMF\_UBIQUITIN\_CONJUGATING\_ENZYME\_BINDING |  | 23 | -0.25 | -0.80 | 0.779 | 0.964 | 1.000 | 2415 | tags=26%, list=19%, signal=32% |
| 373 | GOMF\_PHOSPHATIDYLINOSITOL\_4\_PHOSPHATE\_BINDING |  | 21 | -0.26 | -0.80 | 0.745 | 0.966 | 1.000 | 1704 | tags=19%, list=13%, signal=22% |
| 374 | GOMF\_BETA\_TUBULIN\_BINDING |  | 38 | -0.22 | -0.80 | 0.787 | 0.971 | 1.000 | 1917 | tags=16%, list=15%, signal=18% |
| 375 | GOMF\_PROTEIN\_KINASE\_C\_BINDING |  | 48 | -0.21 | -0.79 | 0.799 | 0.977 | 1.000 | 3199 | tags=23%, list=25%, signal=30% |
| 376 | GOMF\_HYALURONIC\_ACID\_BINDING |  | 21 | -0.25 | -0.79 | 0.770 | 0.980 | 1.000 | 1463 | tags=14%, list=11%, signal=16% |
| 377 | GOMF\_LIGAND\_GATED\_CALCIUM\_CHANNEL\_ACTIVITY |  | 22 | -0.25 | -0.79 | 0.805 | 0.978 | 1.000 | 2558 | tags=27%, list=20%, signal=34% |
| 378 | GOMF\_DEACETYLASE\_ACTIVITY |  | 30 | -0.23 | -0.78 | 0.795 | 0.982 | 1.000 | 1661 | tags=17%, list=13%, signal=19% |
| 379 | GOMF\_TUBULIN\_BINDING |  | 276 | -0.16 | -0.78 | 0.965 | 0.981 | 1.000 | 2650 | tags=20%, list=21%, signal=24% |
| 380 | GOMF\_PHOSPHATIDYLINOSITOL\_PHOSPHATE\_BINDING |  | 128 | -0.17 | -0.78 | 0.910 | 0.983 | 1.000 | 2327 | tags=17%, list=18%, signal=21% |
| 381 | GOMF\_3\_5\_EXONUCLEASE\_ACTIVITY |  | 41 | -0.21 | -0.78 | 0.814 | 0.980 | 1.000 | 3512 | tags=34%, list=27%, signal=47% |
| 382 | GOMF\_PROTEIN\_PHOSPHATASE\_ACTIVATOR\_ACTIVITY |  | 15 | -0.27 | -0.78 | 0.735 | 0.979 | 1.000 | 3348 | tags=40%, list=26%, signal=54% |
| 383 | GOMF\_CORECEPTOR\_ACTIVITY |  | 40 | -0.21 | -0.78 | 0.844 | 0.979 | 1.000 | 2863 | tags=23%, list=22%, signal=29% |
| 384 | GOMF\_PRENYLTRANSFERASE\_ACTIVITY |  | 16 | -0.27 | -0.77 | 0.783 | 0.982 | 1.000 | 3152 | tags=38%, list=24%, signal=50% |
| 385 | GOMF\_POTASSIUM\_ION\_TRANSMEMBRANE\_TRANSPORTER\_ACTIVITY |  | 108 | -0.18 | -0.77 | 0.919 | 0.983 | 1.000 | 2925 | tags=24%, list=23%, signal=31% |
| 386 | GOMF\_INORGANIC\_ANION\_TRANSMEMBRANE\_TRANSPORTER\_ACTIVITY |  | 121 | -0.17 | -0.77 | 0.922 | 0.984 | 1.000 | 4385 | tags=37%, list=34%, signal=56% |
| 387 | GOMF\_LIPASE\_ACTIVATOR\_ACTIVITY |  | 15 | -0.27 | -0.77 | 0.786 | 0.987 | 1.000 | 2810 | tags=40%, list=22%, signal=51% |
| 388 | GOMF\_OXIDOREDUCTASE\_ACTIVITY\_ACTING\_ON\_PAIRED\_DONORS\_WITH\_INCORPORATION\_OR\_REDUCTION\_OF\_MOLECULAR\_OXYGEN\_NAD\_P\_H\_AS\_ONE\_DONOR\_AND\_INCORPORATION\_OF\_ONE\_ATOM\_OF\_OXYGEN |  | 38 | -0.21 | -0.77 | 0.813 | 0.985 | 1.000 | 3320 | tags=32%, list=26%, signal=42% |
| 389 | GOMF\_RNA\_CAP\_BINDING |  | 16 | -0.27 | -0.76 | 0.777 | 0.984 | 1.000 | 1938 | tags=31%, list=15%, signal=37% |
| 390 | GOMF\_PDZ\_DOMAIN\_BINDING |  | 70 | -0.19 | -0.76 | 0.885 | 0.984 | 1.000 | 3622 | tags=34%, list=28%, signal=47% |
| 391 | GOMF\_POTASSIUM\_CHANNEL\_ACTIVITY |  | 83 | -0.18 | -0.76 | 0.902 | 0.992 | 1.000 | 2925 | tags=24%, list=23%, signal=31% |
| 392 | GOMF\_MONOCARBOXYLIC\_ACID\_TRANSMEMBRANE\_TRANSPORTER\_ACTIVITY |  | 39 | -0.21 | -0.75 | 0.858 | 0.992 | 1.000 | 3004 | tags=26%, list=23%, signal=33% |
| 393 | GOMF\_PHOSPHATIDYLINOSITOL\_BISPHOSPHATE\_BINDING |  | 79 | -0.18 | -0.75 | 0.903 | 0.990 | 1.000 | 2684 | tags=19%, list=21%, signal=24% |
| 394 | GOMF\_PHOSPHATIDYLINOSITOL\_KINASE\_ACTIVITY |  | 16 | -0.26 | -0.75 | 0.801 | 0.988 | 1.000 | 5505 | tags=63%, list=43%, signal=109% |
| 395 | GOMF\_CYTOSKELETAL\_MOTOR\_ACTIVITY |  | 85 | -0.18 | -0.75 | 0.940 | 0.991 | 1.000 | 2122 | tags=15%, list=16%, signal=18% |
| 396 | GOMF\_PROTEIN\_SERINE\_THREONINE\_KINASE\_INHIBITOR\_ACTIVITY |  | 25 | -0.23 | -0.75 | 0.860 | 0.990 | 1.000 | 3546 | tags=32%, list=27%, signal=44% |
| 397 | GOMF\_RIBOSOME\_BINDING |  | 54 | -0.19 | -0.75 | 0.904 | 0.988 | 1.000 | 4036 | tags=37%, list=31%, signal=54% |
| 398 | GOMF\_ADRENERGIC\_RECEPTOR\_BINDING |  | 17 | -0.25 | -0.75 | 0.794 | 0.986 | 1.000 | 4690 | tags=53%, list=36%, signal=83% |
| 399 | GOMF\_CHEMOKINE\_BINDING |  | 33 | -0.21 | -0.74 | 0.882 | 0.996 | 1.000 | 1295 | tags=12%, list=10%, signal=13% |
| 400 | GOMF\_PHOSPHATIDYLINOSITOL\_3\_5\_BISPHOSPHATE\_BINDING |  | 20 | -0.23 | -0.73 | 0.839 | 0.995 | 1.000 | 1185 | tags=10%, list=9%, signal=11% |
| 401 | GOMF\_MAP\_KINASE\_KINASE\_ACTIVITY |  | 17 | -0.25 | -0.73 | 0.841 | 0.994 | 1.000 | 3203 | tags=35%, list=25%, signal=47% |
| 402 | GOMF\_ABC\_TYPE\_TRANSPORTER\_ACTIVITY |  | 41 | -0.20 | -0.73 | 0.864 | 0.994 | 1.000 | 4250 | tags=39%, list=33%, signal=58% |
| 403 | GOMF\_PASSIVE\_TRANSMEMBRANE\_TRANSPORTER\_ACTIVITY |  | 344 | -0.14 | -0.73 | 0.997 | 0.992 | 1.000 | 4412 | tags=34%, list=34%, signal=50% |
| 404 | GOMF\_HSP70\_PROTEIN\_BINDING |  | 39 | -0.20 | -0.73 | 0.890 | 0.991 | 1.000 | 3730 | tags=33%, list=29%, signal=47% |
| 405 | GOMF\_DYNEIN\_INTERMEDIATE\_CHAIN\_BINDING |  | 21 | -0.23 | -0.73 | 0.856 | 0.991 | 1.000 | 2128 | tags=24%, list=16%, signal=28% |
| 406 | GOMF\_ACTIVE\_ION\_TRANSMEMBRANE\_TRANSPORTER\_ACTIVITY |  | 202 | -0.15 | -0.73 | 0.993 | 0.989 | 1.000 | 3015 | tags=24%, list=23%, signal=31% |
| 407 | GOMF\_ALPHA\_TUBULIN\_BINDING |  | 30 | -0.21 | -0.72 | 0.880 | 0.989 | 1.000 | 1917 | tags=20%, list=15%, signal=23% |
| 408 | GOMF\_GLUTAMATE\_RECEPTOR\_BINDING |  | 34 | -0.20 | -0.72 | 0.901 | 0.990 | 1.000 | 4225 | tags=38%, list=33%, signal=57% |
| 409 | GOMF\_PROTEIN\_LIPID\_COMPLEX\_BINDING |  | 25 | -0.22 | -0.72 | 0.864 | 0.987 | 1.000 | 1778 | tags=20%, list=14%, signal=23% |
| 410 | GOMF\_EPHRIN\_RECEPTOR\_BINDING |  | 28 | -0.21 | -0.72 | 0.873 | 0.989 | 1.000 | 4073 | tags=39%, list=32%, signal=57% |
| 411 | GOMF\_POLYUBIQUITIN\_MODIFICATION\_DEPENDENT\_PROTEIN\_BINDING |  | 35 | -0.20 | -0.71 | 0.917 | 0.995 | 1.000 | 983 | tags=11%, list=8%, signal=12% |
| 412 | GOMF\_ALPHA\_ACTININ\_BINDING |  | 24 | -0.22 | -0.71 | 0.884 | 0.993 | 1.000 | 3862 | tags=33%, list=30%, signal=47% |
| 413 | GOMF\_CYCLIN\_BINDING |  | 28 | -0.21 | -0.71 | 0.893 | 0.991 | 1.000 | 2601 | tags=21%, list=20%, signal=27% |
| 414 | GOMF\_EXORIBONUCLEASE\_ACTIVITY |  | 30 | -0.20 | -0.69 | 0.905 | 1.000 | 1.000 | 2498 | tags=23%, list=19%, signal=29% |
| 415 | GOMF\_UBIQUITIN\_SPECIFIC\_PROTEASE\_BINDING |  | 17 | -0.23 | -0.69 | 0.872 | 1.000 | 1.000 | 3874 | tags=35%, list=30%, signal=50% |
| 416 | GOMF\_SYNTAXIN\_BINDING |  | 52 | -0.18 | -0.68 | 0.944 | 1.000 | 1.000 | 4700 | tags=40%, list=36%, signal=63% |
| 417 | GOMF\_CARBOHYDRATE\_DERIVATIVE\_TRANSMEMBRANE\_TRANSPORTER\_ACTIVITY |  | 40 | -0.19 | -0.68 | 0.938 | 1.000 | 1.000 | 1992 | tags=18%, list=15%, signal=21% |
| 418 | GOMF\_RNA\_METHYLTRANSFERASE\_ACTIVITY |  | 45 | -0.18 | -0.67 | 0.947 | 1.000 | 1.000 | 2879 | tags=22%, list=22%, signal=29% |
| 419 | GOMF\_ISOPRENOID\_BINDING |  | 29 | -0.20 | -0.67 | 0.943 | 1.000 | 1.000 | 2773 | tags=24%, list=21%, signal=31% |
| 420 | GOMF\_LIPOPROTEIN\_PARTICLE\_RECEPTOR\_ACTIVITY |  | 16 | -0.24 | -0.66 | 0.891 | 1.000 | 1.000 | 893 | tags=13%, list=7%, signal=13% |
| 421 | GOMF\_EPHRIN\_RECEPTOR\_ACTIVITY |  | 16 | -0.23 | -0.65 | 0.910 | 1.000 | 1.000 | 3361 | tags=38%, list=26%, signal=51% |
| 422 | GOMF\_NEUROTRANSMITTER\_RECEPTOR\_ACTIVITY\_INVOLVED\_IN\_REGULATION\_OF\_POSTSYNAPTIC\_MEMBRANE\_POTENTIAL |  | 45 | -0.17 | -0.64 | 0.957 | 1.000 | 1.000 | 4259 | tags=38%, list=33%, signal=56% |
| 423 | GOMF\_SIALIC\_ACID\_BINDING |  | 16 | -0.22 | -0.64 | 0.915 | 1.000 | 1.000 | 3412 | tags=25%, list=26%, signal=34% |
| 424 | GOMF\_AMINO\_ACID\_BINDING |  | 48 | -0.17 | -0.64 | 0.973 | 1.000 | 1.000 | 3694 | tags=29%, list=29%, signal=41% |
| 425 | GOMF\_EXCITATORY\_EXTRACELLULAR\_LIGAND\_GATED\_ION\_CHANNEL\_ACTIVITY |  | 29 | -0.18 | -0.62 | 0.972 | 1.000 | 1.000 | 2976 | tags=28%, list=23%, signal=36% |
| 426 | GOMF\_CALCIUM\_DEPENDENT\_PHOSPHOLIPID\_BINDING |  | 35 | -0.17 | -0.62 | 0.960 | 1.000 | 1.000 | 5134 | tags=49%, list=40%, signal=80% |
| 427 | GOMF\_POSTSYNAPTIC\_NEUROTRANSMITTER\_RECEPTOR\_ACTIVITY |  | 59 | -0.15 | -0.62 | 0.991 | 1.000 | 1.000 | 4990 | tags=44%, list=39%, signal=72% |
| 428 | GOMF\_ALKALI\_METAL\_ION\_BINDING |  | 16 | -0.21 | -0.61 | 0.939 | 1.000 | 1.000 | 4684 | tags=56%, list=36%, signal=88% |
| 429 | GOMF\_CYTOKINE\_RECEPTOR\_ACTIVITY |  | 88 | -0.14 | -0.61 | 0.995 | 1.000 | 1.000 | 3700 | tags=27%, list=29%, signal=38% |
| 430 | GOMF\_SIGNALING\_RECEPTOR\_COMPLEX\_ADAPTOR\_ACTIVITY |  | 45 | -0.16 | -0.60 | 0.988 | 1.000 | 1.000 | 2133 | tags=18%, list=17%, signal=21% |
| 431 | GOMF\_TRANSFORMING\_GROWTH\_FACTOR\_BETA\_BINDING |  | 18 | -0.20 | -0.60 | 0.970 | 1.000 | 1.000 | 2702 | tags=22%, list=21%, signal=28% |
| 432 | GOMF\_ANKYRIN\_BINDING |  | 17 | -0.21 | -0.60 | 0.944 | 1.000 | 1.000 | 3035 | tags=24%, list=24%, signal=31% |
| 433 | GOMF\_HEPARAN\_SULFATE\_PROTEOGLYCAN\_BINDING |  | 17 | -0.20 | -0.59 | 0.960 | 1.000 | 1.000 | 5158 | tags=53%, list=40%, signal=88% |
| 434 | GOMF\_TELOMERASE\_RNA\_BINDING |  | 17 | -0.20 | -0.59 | 0.973 | 1.000 | 1.000 | 3055 | tags=29%, list=24%, signal=38% |
| 435 | GOMF\_ACTININ\_BINDING |  | 32 | -0.17 | -0.57 | 0.977 | 1.000 | 1.000 | 3035 | tags=22%, list=24%, signal=29% |
| 436 | GOMF\_ACETYLCHOLINE\_RECEPTOR\_ACTIVITY |  | 20 | -0.18 | -0.57 | 0.974 | 1.000 | 1.000 | 2662 | tags=25%, list=21%, signal=31% |
| 437 | GOMF\_NUCLEOTIDE\_TRANSMEMBRANE\_TRANSPORTER\_ACTIVITY |  | 16 | -0.20 | -0.56 | 0.970 | 1.000 | 1.000 | 3972 | tags=38%, list=31%, signal=54% |
| 438 | GOMF\_P\_TYPE\_TRANSMEMBRANE\_TRANSPORTER\_ACTIVITY |  | 18 | -0.18 | -0.55 | 0.978 | 1.000 | 1.000 | 2485 | tags=22%, list=19%, signal=27% |
| 439 | GOMF\_PATTERN\_RECOGNITION\_RECEPTOR\_ACTIVITY |  | 21 | -0.18 | -0.55 | 0.984 | 1.000 | 1.000 | 29 | tags=5%, list=0%, signal=5% |
| 440 | GOMF\_SNARE\_BINDING |  | 81 | -0.13 | -0.54 | 0.998 | 1.000 | 1.000 | 4700 | tags=38%, list=36%, signal=60% |
| 441 | GOMF\_HISTONE\_KINASE\_ACTIVITY |  | 15 | -0.17 | -0.49 | 0.986 | 1.000 | 1.000 | 4137 | tags=33%, list=32%, signal=49% |
| 442 | GOMF\_PROTEASOME\_BINDING |  | 16 | -0.17 | -0.48 | 0.989 | 1.000 | 1.000 | 4716 | tags=44%, list=37%, signal=69% |
| 443 | GOMF\_PHOSPHOLIPASE\_A2\_ACTIVITY |  | 23 | -0.15 | -0.48 | 0.996 | 1.000 | 1.000 | 3638 | tags=30%, list=28%, signal=42% |
| 444 | GOMF\_FATTY\_ACID\_LIGASE\_ACTIVITY |  | 16 | -0.16 | -0.47 | 0.998 | 1.000 | 1.000 | 4732 | tags=44%, list=37%, signal=69% |
| 445 | GOMF\_ACID\_THIOL\_LIGASE\_ACTIVITY |  | 22 | -0.15 | -0.46 | 0.998 | 1.000 | 1.000 | 4732 | tags=41%, list=37%, signal=64% |
| 446 | GOMF\_STEROL\_TRANSPORTER\_ACTIVITY |  | 30 | -0.13 | -0.44 | 0.997 | 0.999 | 1.000 | 4020 | tags=27%, list=31%, signal=39% |
Table: Gene sets enriched in phenotype **normal (10 samples) **[plain text format]****
